# Supplementary material for: Cohort profile: the eLIXIR Partnership—a maternity–child data linkage for life course research in South London, UK
Source: BMJ Open. 2020 Oct 6;10(10):e039583. doi: 10.1136/bmjopen-2020-039583 (PMC7539583; doi:10.1136/bmjopen-2020-039583)

BMJ Open is committed to open peer review. As part of this commitment we make the peer review history of every article we publish publicly available.

When an article is published we post the peer reviewers' comments and the authors' responses online. We also post the versions of the paper that were used during peer review. These are the versions that the peer review comments apply to.

The versions of the paper that follow are the versions that were submitted during the peer review process. They are not the versions of record or the final published versions. They should not be cited or distributed as the published version of this manuscript.

BMJ Open is an open access journal and the full, final, typeset and author-corrected version of record of the manuscript is available on our site with no access controls, subscription charges or pay-per-view fees (http://bmjopen.bmj.com).

If you have any questions on BMJ Open's open peer review process please email info.bmjopen@bmj.com

BMJ Open

Cohort Profile: The eLIXIR Partnership - a maternity-child data linkage for Life Course Research in South London.

Journal:	*BMJ Open*
Manuscript ID	bmjopen-2020-039583
Article Type:	Cohort profile
Date Submitted by the Author:	20-Apr-2020

Complete List of Authors:	Carson, Lauren; Institute of Psychiatry Psychology and Neuroscience, Psychological Medicine<br>Azmi, Borscha; Institute of Psychiatry Psychology and Neuroscience, Psychological Medicine<br>Jewell, Amelia; South London and Maudsley NHS Foundation Trust, NIHR Maudsley Biomedical Research Centre<br>Taylor, Clare; Women's College Hospital, Women's College Research Institute<br>Flynn, Angela; King's College London, Women and Children's Health<br>Gill, Carolyn; King's College London, Women and Children's Health; Guy's and Saint Thomas' Hospitals NHS Trust, Women's Health Academic Centre KHP<br>Broadbent, Matthew; South London and Maudsley NHS Foundation Trust, NIHR Maudsley Biomedical Research Centre<br>Howard, Louise; Institute of Psychiatry Psychology and Neuroscience, Section of Women's Health; South London and Maudsley NHS Foundation Trust, NIHR Maudsley Biomedical Research Centre<br>Stewart, Robert; Institute of Psychiatry Psychology and Neuroscience, Psychological Medicine; South London and Maudsley NHS Foundation Trust, NIHR Maudsley Biomedical Research Centre<br>Poston, Lucilla; King's College London, Department of Women and Children's Health

Keywords:	OBSTETRICS, EPIDEMIOLOGY, PSYCHIATRY

SCHOLARONE™
Manuscripts

I, the Submitting Author has the right to grant and does grant on behalf of all authors of the Work (as defined in the below author licence), an exclusive licence and/or a non-exclusive licence for contributions from authors who are: i) UK Crown employees; ii) where BMJ has agreed a CC-BY licence shall apply, and/or iii) in accordance with the terms applicable for US Federal Government officers or employees acting as part of their official duties; on a worldwide, perpetual, irrevocable, royalty-free basis to BMJ Publishing Group Ltd ("BMJ") its licensees and where the relevant Journal is co-owned by BMJ to the co-owners of the Journal, to publish the Work in this journal and any other BMJ products and to exploit all rights, as set out in our licence.

The Submitting Author accepts and understands that any supply made under these terms is made by BMJ to the Submitting Author unless you are acting as an employee on behalf of your employer or a postgraduate student of an affiliated institution which is paying any applicable article publishing charge ("APC") for Open Access articles. Where the Submitting Author wishes to make the Work available on an Open Access basis (and intends to pay the relevant APC), the terms of reuse of such Open Access shall be governed by a Creative Commons licence – details of these licences and which Creative Commons licence will apply to this Work are set out in our licence referred to above.

Other than as permitted in any relevant BMJ Author's Self Archiving Policies, I confirm this Work has not been accepted for publication elsewhere, is not being considered for publication elsewhere and does not duplicate material already published. I confirm all authors consent to publication of this Work and authorise the granting of this licence.

For peer review only

Cohort Profile: The eLIXIR Partnership - a maternity-child data linkage for Life Course Research in South London.

Corresponding Author: Lauren E Carson, Department of Psychological Medicine, Institute of Psychiatry, Psychology and Neuroscience, King's College London, PO84, 16 De Crespigny Park, London, SE5 8AF, UK; lauren.carson@kcl.ac.uk

Borscha Azmi, Department of Psychological Medicine, Institute of Psychiatry, Psychology and Neuroscience, King's College London, UK

Amelia Jewell, NIHR Maudsley Biomedical Research Centre, South London and Maudsley NHS Foundation Trust, London, UK

Clare Taylor, Women's College Research Institute, Women's College Hospital, Toronto, Canada

Angela C Flynn, Department of Women and Children's Health, King's College London, UK

Carolyn Gill, Department of Women and Children's Health, King's College London, UK and Women's Health Academic Centre KHP, Guy's and St Thomas' NHS Foundation Trust, UK

Matthew Broadbent, NIHR Maudsley Biomedical Research Centre, South London and Maudsley NHS Foundation Trust, London, UK

Louise M Howard, Section of Women's Mental Health, Institute of Psychiatry, Psychology and Neuroscience, King's College London, UK and NIHR Maudsley Biomedical Research Centre, South London and Maudsley NHS Foundation Trust, London,

Robert Stewart, Department of Psychological Medicine, Institute of Psychiatry, Psychology and Neuroscience, King's College London, UK and NIHR Maudsley Biomedical Research Centre, South London and Maudsley NHS Foundation Trust, London, UK

Lucilla Poston, Department of Women and Children's Health, King's College London, UK

On behalf of the eLIXIR Partnership.

Word Count: 3,826

Key words: Pregnancy, cohort, longitudinal, maternity, infant, life course

For peer review only

ABSTRACT

Purpose

Linked maternity, neonatal and maternal mental health records were created to support research into the early life origins of physical and mental health, in mothers and children. The Early Life Cross Linkage in Research (eLIXIR) Partnership was developed in 2018, generating a repository of real-time, anonymised, structured data derived from the electronic health record systems of two Acute and one Mental Health Care NHS Provider in South London. We present early descriptive data for the database, and the robust data security and governance structures, and describe the intended expansion of the database from its original development. Additionally, we report details of the accompanying eLIXIR Research Tissue Bank of maternal and neonatal blood samples.

Participants

Descriptive data were generated from the eLIXIR database from 1 October 2018 to 30 June 2019. Over 17,000 electronic patient records were included from 10,207 women, of whom 1,184 also accessed mental health care.

Findings to date

10,207 women accessed antenatal care from the two NHS maternity services, with 8,405 deliveries (8,772 infants). This diverse, inner city maternity service population were born in over 170 countries with an ethnic profile of 46.1% White, 19.1% Black, 7.0% Asian, 4.1% Mixed and 4.1% other. Of the 10,207 women, 11.6% had a clinical record in mental health services with 3.0% being treated during their pregnancy. This first data extract included 947 infants treated in the neonatal intensive care unit (NICU), of whom 19.1% were postnatal transfers from external healthcare providers.

Future plans

The eLIXIR database will grow by ~14,000 new maternity cases annually, in addition to providing child follow-up data. Additional data will supplement the current linkage from other local and national resources including primary care and hospital inpatient data for mothers and their children.

For peer review only - http://bmjopen.bmj.com/site/about/guidelines.xhtml

STRENGTHS AND LIMITATIONS

- eLIXIR is a unique population-based database incorporating clinical data from maternity, neonatal and mental health records enabling life course studies of physical and mental health in a large, diverse, inner city, UK population.
- Finding of studies undertaken using eLIXIR has implications for local health care improvement but also has the potential to provide evidence to influence healthcare in similar national/global settings.
- Missingness and inaccuracy in all routine/administrative clinical databases will be a key limitation to this database
- The representativeness of the cohort to the UK population is limited to mixed, inner-urban catchments.

For peer review only

INTRODUCTION

Investment in health in the earliest stages of life is increasingly recognised as a means to improve the life course of health; beginning *in utero*, through to infancy, childhood and into adulthood [1 2]. Much of the supporting evidence underpinning a 'life course approach' to disease prevention from pregnancy and infancy onwards has been accrued from large birth cohorts [3-6]; however, directly recruited cohorts are by definition drawn from individuals recruited over a pre-specified and limited time period and thereby become rapidly outdated as temporal shifts in population demography, lifestyle and ethnicity occur. Moreover, facility-centred follow-up is expensive and difficult to sustain [7 8]. Sample attrition is common and can introduce significant methodological biases that may affect the validity of investigations into novel risk outcomes [9]. In addition, direct recruitment may result in cohorts with limited representation of the target population because of selective inclusion. Population-based registries also offer insight into rarer diseases and outcomes, not feasible in current research cohorts.

Linked administrative data are increasingly used to provide evidence to guide policy and clinical management [10-12]. The longitudinal nature of such case registers, their size, and coverage of defined populations provide an increasingly attractive alternative to the study of birth cohorts for defining the early life exposures that contribute to the population burden of physical and mental health disorders. These can provide longitudinal information on large numbers of women and children, as well as the potential for linkage with a widening portfolio of available local and national datasets to follow health from birth to adulthood (https://digital.nhs.uk/data-and-information/data-collections-and-data-sets/data-sets). Both inclusion and attrition bias of traditional birth cohorts can be overcome through routine comprehensive health records, as these can capture rich clinical data in a given population on all women receiving antenatal care, and their infants [13]. Whilst well established in Scandinavian countries, national birth registries in the UK have not been widely used in linkage programmes using infant and childhood data, although population registry data from Scotland have provided information on relationships between maternal and neonatal outcomes which has informed clinical guidelines in the UK and beyond [14]. Several recent linkages of clinical maternity data have nonetheless shown the feasibility of the approach, and usefulness, for example in aligning hospital maternity data with birth registration datasets and providing insight into relationships between adolescent infection and prematurity [15-23].

It is well established that maternal physical and mental wellbeing in pregnancy and the postpartum period can strongly influence neonatal outcome and the physical and mental health of the child [24-27].

For peer review only - http://bmjopen.bmj.com/site/about/guidelines.xhtml

To our knowledge, no clinical data linkages in maternity or neonatal services have to date incorporated both physical and mental health data to interrogate these associations at a population level. The eLIXIR Partnership has been developed to address these relationships from early pregnancy, the perinatal period and beyond into later life. Funded by the Medical Research Council (MRC) in 2017, the partnership is a multidisciplinary academic collaboration which aims to combine maternal, infant and child health data into a single resource to allow information from large numbers of mothers, babies and children to be investigated over an unlimited time period. The intention is to provide a naturally accumulating database to support investigations into associations between physical and mental health in mother and child.

The eLIXIR Partnership provides a mechanism through which research datasets can be linked to clinical records, under appropriate and approved levels of anonymity and data security. An added benefit is the potential to incorporate data from multiple sources, e.g. health, environment, social, and education. There are, however, important ethical and legal considerations, as well as technical security requirements, if linkages are to be performed between sources of routinely collected clinical data, and exemption from individual consent to be permissible [28].

Another aspect of the eLIXIR Partnership is the eLIXIR Research Tissue Bank established to link the routinely collected maternal and neonatal clinical data with biological samples. This has an advantage over static cohort studies by providing a “dynamic” collection of samples, enabling identification of population trends, and influences of new clinical interventions. The provision of samples from women attending antenatal care will provide a unique biobank to address mechanisms of common and rarer complications in pregnancy and in neonatal life, and their consequences for the longer-term health of the mother and child. Common complications will include gestational diabetes, mental illness, prematurity and pre-eclampsia. Similarly, by the provision of samples from neonatal intensive care, eLIXIR will contribute to a better understanding of neonatal morbidity and mortality.

With records of over 14,000 individual births per year, eLIXIR has the potential to become one of the largest mother-infant-child datasets in Europe. This has been facilitated by King’s Health Partners (KHP): an Academic Health Sciences Centre which brings together one academic institution (King’s College London (KCL)) and three NHS Foundation Trusts (Guys and St Thomas’ NHS Foundation Trust (GSTT), King’s College Hospital NHS Foundation Trust (KCH) and South London and Maudsley NHS Foundation Trust (SLaM)).

For peer review only

This manuscript details the technical and procedural elements in place to safeguard the legal and ethical rights of service users during the development and use of the eLIXIR database, and to present the demographic profile of the eLIXIR population. Both technical and procedural elements draw strongly on experience gained in setting up the Clinical Record Interactive Search (CRIS) data resource at the Maudsley National Institute for Health Research (NIHR) Biomedical Research Centre (BRC) [29-31].

Benefits of the system

Large data linkage platforms, such as that created by the eLIXIR Partnership, provide a unique data warehouse through which important epidemiological questions can be asked – in the case of eLIXIR, within a large and diverse inner-city population. The ability to conduct these linkages allows not only the collection of a wide range of longitudinal health and social data, but also the capacity to support life course data analysis. The potential benefits arising from the use of clinical record 'big data' have been widely reported, and research databases such as eLIXIR are likely to increase in number due to the powerful and cost-effective nature of this research method [32 33]. We believe this is one of the first longitudinal research programmes from early pregnancy onward using routinely collected clinical data which does not rely on a recruited cohort of participants.

COHORT DESCRIPTION

Data sources

Maternity and neonatal data was obtained from GSTT and KCH, and mental health data from SLaM. These Trusts provide healthcare services across central and outer London locations, and include seven individual mental health and physical healthcare hospital sites and many community sites serving over 4.3 million patients per year.

Maternity, birth and neonatal intensive care data

The BadgerNet Platform (CleverMed) for routine clinical data is used extensively across the UK to create electronic patient records, with databases that capture maternity, neonatal intensive care, neonatal transport, paediatric intensive care, neurology referrals and adult intensive care data [34]. Within GSTT and KCH the BadgerNet platforms are used for recording maternity data and neonatal intensive care data (NICU). The BadgerNet system records clinical records on a Single Care Record system which is nationally hosted. Although feasible within the BadgerNet system, linkage between maternity and neonatal data is not routinely conducted.

For peer review only

Mental health data

Clinical records have been fully electronic across all SLaM NHS Trust mental health services since April 2006, using the bespoke electronic Patient Journey System (ePJS) which incorporated legacy data from earlier service specific electronic health records (EHRs). The Clinical Record Interactive Service (CRIS) platform[31] was developed in 2007–2008 and consists of a series of data-processing pipelines which both structure and de-identify PJS fields, rendering effectively anonymised data from the full clinical record available at the researcher interface, with search and database assembly functionality facilitated by a front end, designed for non-technical use. The de-identifying process and its effectiveness, including the masking of identifying information in open-text fields and the generation of a pseudonymised identifier (CRIS ID), have been previously described [30]. The wider patient-led oversight and security models for CRIS have not changed significantly since it was established [29-31]. Ethical approval was obtained for CRIS as an anonymised database for secondary analysis (Oxford C Research Ethics Committee, reference 18/SC/0372). In terms of cohort coverage, all SLaM care is represented on CRIS, including Improving Access to Psychological Therapies (IAPT): a large primary care service providing short term psychological therapies accessed through primary care referral.

Data linkage hosting environment

Data for eLIXIR are managed and stored at the Clinical Data Linkage Service (CDLS) at SLaM: an impartial trusted third-party service that provides researchers access to linked clinical data in accordance with the strict governance conditions and processes agreed with relevant data controllers. The CDLS is managed by a small, dedicated team of informaticians, IT, and Information Governance (IG) professionals (https://www.maudsleybrc.nihr.ac.uk/facilities/clinical-record-interactive-search-cris/), and currently hosts a range of datasets already linked with the SLaM CRIS mental health case register (e.g. Hospital Episode Statistics, National Cancer Registry, ONS death certification, Lambeth DataNet primary care records, and National Pupil Database). The backbone of the eLIXIR database consists of a 'master patient index' (Figure 1) allowing data to be robustly linked within an appropriately secure environment according to data specifications.

The CDLS hosts both source data and the master patient index, on behalf of the eLIXIR Partnership, on a secure server within the NHS firewall with role-restricted access. The CDLS additionally provides a data extraction service which meets security requirements, creating bespoke datasets for approved research use. Such derived data are managed by the approved research team and are

For peer review only - http://bmjopen.bmj.com/site/about/guidelines.xhtml

hosted at all times on a dedicated drive within the NHS for analysis in this domain using hosted software already available at SLaM.

Four distinct services are offered by the CDLS as the data processor for the eLIXIR data. First, CDLS provides advice on permissions, approvals and contracts. These include consideration of academic, technical, legal and ethical requirements. Second, CDLS facilitates data linkages either within the CDLS safe haven or via a third party, coordinating the secure transfer of data. Third, the CDLS is responsible for the secure storage of linked data in accordance with predefined information governance and security standards. Fourth, the CDLS, as the custodian for the linked data, prepares and extracts bespoke and prespecified databases for approved eLIXIR projects and provides these to researchers. Therefore, there is no direct access by researchers to the full linked data files, enhancing data protection and confidentiality.

Data linkage procedures and resources

The eLIXIR Partnership uses common identifiers (e.g. hospital number, NHS number, name, and date of birth) to link between maternity, neonatal and mental health clinical data, which is undertaken by CDLS staff, not researchers. Matching is undertaken using deterministic matching techniques on a given set of identifiers. Two records are said to match if all or some of the identifiers are identical, as defined by a hierarchical set of match-ranks. This creates a single master patient index including patient anonyms (mother and child), binary variables for presence/absence on each data source, and key demographics. The source datasets and master patient index are stored by the CDLS behind the NHS firewall and linked data are extracted on a project-by-project basis by a CDLS informatician containing the variables and samples necessary for each study, with a study-specific encrypted anonym which contains completely de-identified data. In this way databases are not stored in linked format. The clinical data from maternity and neonatal services are extracted from BadgerNet systems at both GSTT and KCH by ICT staff at each site. These data are then sent securely to the staff at the SLaM CDLS, and linked, using their common identifiers, with data from the CRIS system to incorporate mental health clinical data, where present, for each patient. This results in a comprehensive data resource to which researchers can apply for extracted data across maternity, neonatal and/or mental health services. Figure 1 details the dataflow for the eLIXIR Partnership database.

For peer review only - http://bmjopen.bmj.com/site/about/guidelines.xhtml

Information governance framework

Results from linkages, current and prior, are stored within the CDLS safe haven, and the CDLS plays a key role in wider governance, supplementing the role of eLIXIR-specific oversight and data security, including the secure handling and storage of identifier fields required for data linkage. Section 251 of the NHS Act 2006 allows the common law duty of confidentiality to be set aside in specific circumstances where anonymised information is not sufficient and where 'opt-in' patient consent is not practical. Opt-out information and details of the project are given to each patient entering maternity and neonatal services, and patients have the option of opting out of the programme at any time. Approval under this legal framework was granted by the Health Research Authority (HRA) Confidentiality Advisory Group (CAG) to the eLIXIR team for all the above linkages, which allow data to be available in an identifiable format to a small number of data processing staff in accordance with data sharing contracts between the data provider institutions (HRA CAG Ref: 18/CAG/0040). Activity for projects using linked data sets held by eLIXIR is audited by the eLIXIR Oversight Committee, helping to ensure that the researcher's project requirements (e.g. clinical research, surveillance, service improvement or audit) are met, and that projects progress within the agreed policy and practice framework. The primary role of the Committee is to provide the operational management of eLIXIR as identified in the eLIXIR Security Model and protocol for the eLIXIR Research Tissue Bank. In so doing, the Committee seeks to promote the scientific and ethical principles that should govern the use of eLIXIR data and seeks to represent stakeholders [who include the KHP Caldicott Guardians (individuals responsible for research governance at each NHS Trust site), service users, clinical professionals and academics] and reflect their views and interests.

Public and patient involvement

Public and patient involvement (PPI) is ensured in the decision-making process of approving projects using linked data held by eLIXIR, and the eLIXIR Oversight Committee reviews and approves all projects using eLIXIR data.

Research Tissue Bank

The eLIXIR Research Tissue Bank is a prospective biobank of samples from pregnant women and infants being treated by GSTT. The Research Tissue Bank is integrated into the King's College London HTA governance structure with active recruitment and collection of samples. All pregnant women aged over 16 years who are willing and able to give informed consent, and infants admitted to the neonatal unit that have parental consent, are eligible for inclusion in the eLIXIR Research Tissue Bank (Cambridge East Research Ethics Committee, reference 18/EE/0120).

For peer review only - http://bmjopen.bmj.com/site/about/guidelines.xhtml

Maternal blood sample collection

Eligible women are recruited at the time of routine antenatal care venepuncture (11-15 weeks' gestation or later transfer of care or later antenatal care attendance) at GSTT. Women who agree to participate give written informed consent and an extra blood sample is collected at the same time as routine venepuncture, maximum volume 12ml (2 x 6ml tubes).

Infant blood sample collection

For infants admitted to the neonatal intensive care unit (NICU), blood is drawn for routine tests. Following written informed parental consent, residual blood from routine samples, which otherwise would be discarded, is retained and collected. The samples are processed and stored in a similar manner to the maternal blood samples. Following birth in the community setting (home visits), every infant is offered new-born blood spot screening to exclude metabolic disorders. A health professional pricks the baby's heel to collect 4 drops of blood on a card. For the biobank, an extra card to those taken for clinical purposes is used to collect extra bloodspots from the infant, after the routine spots are collected using the same heel prick. This sample is posted back to the eLIXIR team and stored in the research laboratory prior to transfer to a central storage facility.

All samples are transferred for processing in the research laboratory. After processing, all tubes are labelled with a study specific barcode and entered on to a study specific database (FreezerPro©). The samples are stored in -80°C freezers short-term, before being transported to a central storage facility (NIHR BioResource, Milton Keynes).

FINDINGS TO DATE

Maternal, Birth and Birth Outcomes

From the first data extraction (1 October 2018 - 30 June 2019) 10,207 women accessed antenatal care through GSTT or KCH maternity services with 8,405 deliveries (8,772 infants). This diverse, inner city antenatal population were born in over 170 countries with a heterogeneous distribution across ethnic groups (Table 1). Most were born outside the UK but most reported English as their primary language. Women were booked on average at 11.6 weeks gestation, which is slightly higher than the national guidelines of before 10 weeks [35]. The most common physical conditions experienced were; gynaecological problems (14%), asthma (8%) and pre-existing diabetes (6%). In addition, around one in five reported mental health problems, 3% were recorded as being exposed to female genital mutilation and 4% reported being current smokers at the time of their antenatal appointment. (Table 1)

For peer review only - http://bmjopen.bmj.com/site/about/guidelines.xhtml

Table 1 – Characteristics of women attending antenatal booking appointments at Guy's & St. Thomas' NHS Foundation Trust (GSTT) and King's College Hospital NHS Foundation Trust (KCH) between 1st October 2018 and 30th June 2019.

	GSTT (n=5,700)	*KCH (n=4,507)*	*Across both NHS Trusts (n=10,207)*
Age of mother at booking in years (mean ± SD)	32.7±5.5	32.7±5.6	32.7±5.5
Ethnicity N (%)			
White	2,560 (44.9%)	2,151 (47.7%)	4,711 (46.1%)
Black	862 (15.0%)	1,089 (24.3%)	1,951 (19.1%)
Asian	474 (8.3%)	248 (5.6%)	722 (7.0%)
Mixed	218 (3.8%)	192 (4.3%)	410 (4.1%)
Other	248 (4.4%)	253 (5.6%)	501 (4.9%)
Not Recorded	1,338 (23.4%)	574 (12.7%)	1,912 (18.8%)
Born in the UK (%)	2,150 (37.7%)	2,358 (52.3%)	4,508 (44.1%)
Primary Language English (%).	3,711 (65.1%)	3,474 (77.1%)	7,185 (70.4%)
Gestational age at booking (weeks) (mean ± SD)	11.9±6.4	11.2±6.4	11.6±6.5
Parity (Mean ± SD)	0.7 ±1.1	0.8±1.1	0.8±1.1
Gravida (Mean ± SD)	2.2±1.5	2.4±1.6	2.3±1.6
Prior medical conditions			
Haematological disorder	231 (4.1%)	407 (9.0%)	638 (6.3%)
Thrombosis	57 (1.0%)	35 (0.8%)	90 (0.9%)
Cardiac disorders	103 (1.8%)	103 (2.3%)	206 (2.0%)
Hypertension	108 (2.0%)	85(1.9%)	193 (1.9%)
Renal disorders	155 (2.8%)	135 (3.0%)	290 (2.9%)
Asthma	401 (7.0%)	413 (9.3%)	814 (8.0%)
Lung disorders	14 (0.2%)	10 (0.3%)	24 (0.2%)
Diabetes	375 (6.6%)	257 (5.7%)	627 (6.2%)
Endocrine disorders	307 (5.4%)	205 (4.6%)	512 (5.1%)
Autoimmune disorders	83 (1.4%)	62 (1.4%)	145 (1.4%)
Epilepsy	43 (0.7%)	37 (0.8%)	80 (0.8%)
Neurological disorders	146 (2.6%)	355 (7.9%)	501 (5.0%)
Gastrointestinal disorders	265 (4.6%)	282 (6.2%)	547 (5.4%)

For peer review only - http://bmjopen.bmj.com/site/about/guidelines.xhtml

Gynaecological disorders	742 (13.0%)	673 (15.0%)	1415 (14.1%)
Liver disorders	46 (0.9%)	55 (1.2%)	101 (1.0%)
Bone disorders	38 (0.7%)	59 (1.4%)	97 (0.9%)
Joint disorders	98 (1.7%)	77 (1.7%)	175 (1.8%)
Back problems	173 (3.0%)	375 (8.4%)	548 (5.1%)
Genetic disorders	‡ND	‡ND	‡ND
Congenital abnormalities	29 (0.5%)	27 (0.7%)	56 (0.5%)
Other	85 (1.5%)	121 (2.6%)	206 (2.0%)
Mental health problems (current or historical; self-reported)	988 (13.8%)	1,147 (25.5%)	2,135 (20.9%)
Victim of Female Genital Mutilation (FGM) (%yes)	209 (3.7%)	137 (3.0%)	346 (3.4%)
Smoker at booking (%yes)	195 (3.4%)	199 (4.4%)	394 (3.9%)

‡Not Disclosed (ND). Actual numbers suppressed to reduce risk of statistical disclosure

With regard to birth episodes, twice as many women gave birth at GSTT than KCH with a mean gestational age at delivery of 38.8 weeks, and with 8% born prematurely (<37 completed weeks of gestation) and 3% born very prematurely (<34 weeks' gestation). Of the 8,405 births, 8,051 were singletons, 341 were twin births and 13 were triplets. Around half of deliveries were spontaneous cephalic, around one in five were emergency (or unspecified) caesarean section, and 15% elective caesarean section. Rates of stillbirth and neonatal deaths were 0.6% and 0.4% respectively. The mean birthweight was 3257g with 17% small for gestational age (SGA) and 7% large for gestational age (LGA) (Table 2).

Table 2 – Characteristics of births and birth outcomes at Guy's & St. Thomas' NHS Foundation Trust (GSTT) and King's College Hospital NHS Foundation Trust (KCH) between 1st October 2018 and 30th June 2019.

	GSTT (n=5,697)	*KCH (n=2,708)*	*Across both NHS Trusts (n=8,405)*
Gestation at delivery (weeks)	38.8±2.4	38.9±2.3	38.8±2.4
Very premature (<34 weeks)	193 (3.4%)	91 (3.4%)	284 (3.4%)
Premature (>34 weeks - <37 weeks)	295 (5.2%)	117 (4.3%)	412 (4.9%)
Term (>37 weeks)	5209 (91.4%)	2708 (92.3%)	7709 (91.7%)
Number of infants born (number of pregnancies)			
Singleton	5459 (95.8%)	2592 (95.7%)	8051 (95.8%)

For peer review only - http://bmjopen.bmj.com/site/about/guidelines.xhtml

Twins	227 (4.0%)	114 (4.2%)	341 (4.1%)
Triplets	‡ND	‡ND	13 (0.2%)
Type of delivery			
Breech	32 (0.6%)	16 (0.6%)	48 (0.6%)
Elective Caesarean Section	862 (15.1%)	412 (15.2%)	1274 (15.2%)
Emergency or unspecified Caesarean Section	1132 (19.9%)	461 (17.0%)	1593 (19.0%)
Forceps	462 (8.1%)	180 (6.6%)	642 (7.6%)
Spontaneous Cephalic	2838 (49.8%)	1378 (50.9%)	4216 (50.2%)
Ventouse	371 (6.5%)	257 (9.5%)	628 (7.5%)
Birth outcome			
Live birth	5644 (99.1%)	2680 (99.0%)	8324 (99.0%)
Stillbirth or neonatal death	53 (0.9%)	28 (1.0%)	81 (1.0%)
Birth weight (grams)	3249.1±621.7	3273.3±621.25	3256.9±621.6
Small for Gestational Age (Birth Centile ≤10th)	551 (9.7%)	259 (9.6%)	810 (9.7%)
Large for Gestational Age (Birth Centile≥90th)	551 (6.6%)	172 (6.4%)	549 (6.6%)

‡Not Disclosed (ND). Actual numbers suppressed to reduce risk of statistical disclosure

Neonatal Intensive Care Unit admissions–

Of the 947 infants that had been treated in neonatal intensive care units across both GSTT and KCH, 19.1% were postnatal transfers from an external Trust and 8.7% were in-utero transfers. The main reason for admission was for respiratory disease (28%) followed by preterm birth (22%). The average length of time spent in NICU was 15.8 days. Of outcomes following admission 46% were re-admitted to a postnatal ward, 29% were discharged home, and neonatal death occurred in 4%, the remainder being unknown from the data available (Table 3)

Table 3 - Characteristics of patients admitted to neonatal intensive care unit (NICU) at Guy's & St. Thomas' NHS Foundation Trust (GSTT) and King's College Hospital NHS Foundation Trust (KCH) between 1st October 2018 and 30th June 2019.

	GSTT (n=645)	*KCH (n=302)*	*Total (n=947)*
Gestational age (weeks)	35.2 (±5.0)	35.7 (±4.6)	35.3 (±4.9)
Birthweight (grams)	2541.1 (±1075.1)	2586.4 (±1022.2)	2555.6 (±1051.32)
Number of days on NNU	15.3±24.7	16.9(±26.2)	15.8 (±25.2)

For peer review only - http://bmjopen.bmj.com/site/about/guidelines.xhtml

Number of episodes of care			
1	476 (73.8%)	254 (84.1%)	730 (77.1%)
2	135 (20.9%)	39 (12.9%)	174 (18.4%)
3	‡ND	‡ND	30 (3.2%)
4+	‡ND	‡ND	13 (1.2%)
Readmission (yes)	25 (3.9%)	11 (3.6%)	36 (3.8%)
Consanguineous parents	‡ND	‡ND	11 (1.2%)
Reason for admission			
Preterm Birth	132 (20.5%)	73 (24.2%)	205 (21.6%)
Respiratory disease	172 (26.7%)	90 (29.8%)	262 (27.6%)
Cardiovascular disease	‡ND	‡ND	93 (9.8%)
Infection	11 (1.7%)	12 (4.0%)	23 (2.4%)
Jaundice	29 (4.5%)	11 (3.6%)	40 (4.2%)
Poor Feeding/Weight loss	‡ND	‡ND	21 (2.2%)
Gastro-intestinal disease	‡ND	‡ND	24 (2.5%)
Hypoglycaemia	34 (5.3%)	13 (4.3%)	47 (5.0%)
Convulsions or neurological disease	‡ND	‡ND	12 (1.3%)
Congenital abnormality	‡ND	‡ND	28 (2.3%)
Surgery	67 (10.4%)	27 (8.9%)	94 (9.9%)
Investigation/monitoring	26 (4.0%)	14 (4.6%)	40 (4.3%)
Poor condition at birth	‡ND	‡ND	22 (2.3%)
Other	19 (2.9%)	17 (5.6%)	36 (3.8%)
Maternal drug use	‡ND	‡ND	13 (1.4%)
Maternal smoking (n)	23 (3.6%)	20 (6.6%)	43 (4.5%)
Neonatal outcome			
Ward	281 (43.6%)	152 (50.3%)	433 (45.7%)
Home	181 (28.1%)	97 (32.1%)	278 (29.4%)
Died	27 (4.2%)	13 (4.3%)	40 (4.2%)
Unknown	156 (24.2%)	40 (13.2%)	196 (20.7%)

‡Not Disclosed (ND). Actual numbers suppressed to reduce risk of statistical disclosure

For peer review only - http://bmjopen.bmj.com/site/about/guidelines.xhtml

Mental Health

Of the 10,207 women attending antenatal care as registered in the eLIXIR database, 1,184 had a clinical record in secondary mental health services (SLaM) with 307 women actively being treated at the time of their pregnancy (201 under the care of Improving Access to Psychological Therapies (IAPT)) (Table4).

Table 4 - Mental health data (South London and Maudsley NHS Foundation Trust, SLaM) of women receiving antenatal care at Guy's & St. Thomas' NHS Foundation Trust (GSTT) and King's College Hospital NHS Foundation Trust (KCH) between 1st October 2018 and 30th June 2019. IAPT IN FULL Here

	GSTT (n=5700)	*KCH (n=4507)*	*Across both NHS Trusts (n=10,207)*
Ever received treatment from SLaM	658 (11.5%)	526 (11.7%)	1184 (11.6%)
Under SLaM Care during pregnancy	180 (3.2%)	127 (2.8%)	307 (3.0%)
Received psychological therapy (through IAPT) in pregnancy from SLaM	113 (2.0%)	88 (2.0%)	201 (2.0%)

Research Tissue Bank

Following all necessary governance agreements samples were collected over a period of 3months. A total of 1,293 aliquoted samples (including EDTA, serum and whole blood) from 101 women were stored in the FreezerPro© system.

STRENGTHS AND LIMITATIONS

The eLIXIR Partnership has developed a unique population-based database incorporating clinical data from maternity, neonatal and mental health records. This is supplemented by the eLIXIR Research Tissue Bank. Together this resource will provide the basis for additional linkages to enable life course studies of physical and mental health in a large, diverse, inner city, UK population. This will not only have implications for local health care improvement but also has the potential to provide evidence to influence healthcare in similar national/global settings.

A limitation common to all routine/administrative clinical databases is source data missingness and inaccuracy. This, in contrast, is an advantage of prospective research cohorts. Nonetheless eLIXIR provides opportunity for continuous feedback to the clinical provider on data absence or error. With

For peer review only - http://bmjopen.bmj.com/site/about/guidelines.xhtml

regular meetings we already appraise the Trust IT teams of, for example, duplicate patient data entry and missing data, especially that required for the national Maternity Services Data Set (MSDS). Thus, eLIXIR and other similar research datasets can contribute directly to improved clinical reporting, and hence to better clinical care.

Another potential limitation is loss of data from women who move outside of the catchment area within the index pregnancy and thereafter, although it is our intention to supplement the linkages with data from national Hospital Episode Statistics (HES), to provide information on hospitalised outcomes. As with all administrative clinical datasets, research will be limited to that which can be conducted using information routinely collected in clinical care. The intended incorporation of research datasets offsets this to some extent. Additionally, clinical data entry may involve substantial human error, and data may be absent from the clinical records (i.e. missing). The representativeness of the cohort to the UK population is limited to mixed, inner-urban catchments. In addition, all three NHS Trusts involved in the eLIXIR Partnership incorporate specialisms or local expertise attracting national-level referrals. As a result, data may be skewed to patients with more severe or complex health issues.

Plans for the future

As the eLIXIR database develops, expansions will incorporate health and social care data. The next phase of linkage will comprise a local primary care data resource, Lambeth DataNet (https://www.lambethccg.nhs.uk/your-health/Information-for-patients/lambeth-datanet/Pages/default.aspx), for which all necessary approvals are in place. Beyond-catchment hospitalisation data could be usefully captured, by linkage to national data sources, e.g. HES, as mentioned, which has been incorporated in the CRIS platform[31]. Subject to approval, later linkages will incorporate prescribing and education data, in addition to a broader range of local healthcare information as eLIXIR infants enter age ranges covered by other specialties. Finally, replication of this data-linkage model in other geographical settings in the UK would offer the potential to develop a national data network allowing both larger research cohorts and cross-site replication.

COLLABORATION

We have established a research database of maternity, neonatal and mental health clinical data not only combining maternal physical and mental health clinical data during pregnancy and later neonatal health, but also provides added value through potential for addition of biological measurements (i.e. omics data) from the eLIXIR Tissue Bank samples. The eLIXIR Programme has the

For peer review only - http://bmjopen.bmj.com/site/about/guidelines.xhtml

capacity to continue to grow and develop exponentially, through internal and external collaborations, with small levels of attrition to follow-up and the ability to be used for both common and rare research outcomes. Furthermore, unlike comparable research programmes, the population we sample from is diverse on both an ethnicity and sociodemographic level providing a richness of data which has the potential to lead to exciting research findings.

FURTHER DETAILS

Data sharing statement

Researchers can apply for data access and biomaterial by submitting a research application form to the eLIXIR team. The eLIXIR website provides information on the application process (http://www.guysandstthomasbrc.nihr.ac.uk/microsites/elixir).

Funding declaration

This work was supported by an MRC Partnership Grant [MR/P003060/1].

LC and LH receives salary support from the National Institute for Health Research (NIHR) Applied Research Collaboration South London (NIHR ARC South London) at King's College Hospital NHS Foundation Trust. LC was additionally supported by a Medical Research Council Mental Health Data Pathfinder Award to King's College London. LC, AJ, MB, LH, RS are part-funded by the National Institute for Health Research (NIHR) Biomedical Research Centre at the South London and Maudsley NHS Foundation Trust and King's College London. AF and LP are supported by Tommy's Charity and King's College London. CG and LP are supported by the National Institute for Health Research (NIHR) Biomedical Research Centre at Guy's and St. Thomas' NHS Foundation Trust.

Conflict of interest

RS declares research support received in the last 5 years from Roche, Janssen, GSK and Takeda.

Disclaimer

The views expressed are those of the authors and not necessarily those of the NHS, the NIHR, the MRC or the Department of Health.

Ethics approval

Oxford C REC (18/SC/0086) and Cambridge East REC (18/EE/0120).

For peer review only

Contributorship statement

LC, LH, RS, LP contributed to the conception of this article. LC, BA, AJ, CT, AF, CG, LH, RS, LP were involved in manuscript writing and revision. LC, MB, LP were involved in data analysis and interpretation. All authors read and approved the final manuscript.

For peer review only

References

1. Mikkelsen B, Williams J, Rakovac I, et al. Life course approach to prevention and control of non-communicable diseases. *Bmj* 2019;364:l257. doi: 10.1136/bmj.l257
2. Kuruvilla S, Sadana R, Montesinos EV, et al. A life-course approach to health: synergy with sustainable development goals. *Bull World Health Organ* 2018;96(1):42-50. doi: 10.2471/BLT.17.198358 [published Online First: 2017/11/23]
3. Fraser A, Macdonald-Wallis C, Tilling K, et al. Cohort Profile: the Avon Longitudinal Study of Parents and Children: ALSPAC mothers cohort. *International journal of epidemiology* 2013;42(1):97-110. doi: 10.1093/ije/dys066
4. Jaddoe VW, Mackenbach JP, Moll HA, et al. The Generation R Study: Design and cohort profile. *European journal of epidemiology* 2006;21(6):475-84. doi: 10.1007/s10654-006-9022-0
5. Wright J, Small N, Raynor P, et al. Cohort Profile: The Born in Bradford multi-ethnic family cohort study. *International journal of epidemiology* 2012;42(4):978-91. doi: 10.1093/ije/dys112
6. Sebert S, Lowry E, Aumüller N, et al. Cohort Profile: The DynaHEALTH consortium – a European consortium for a life-course bio-psychosocial model of healthy ageing of glucose homeostasis. *International journal of epidemiology* 2019;48(4):1051-51k. doi: 10.1093/ije/dyz056
7. Overy CR, LA; Tansey, EM, ed. History of the Avon Longitudinal Study of Parents and Children , C 1980-2000. History of the Avon Longitudinal Study of Parents and Children, C 1980-2000; 2011. Queen Mary, University of London.
8. Downs JM, Ford T, Stewart R, et al. An approach to linking education, social care and electronic health records for children and young people in South London: a linkage study of child and adolescent mental health service data. *BMJ Open* 2019;9(1):e024355. doi: 10.1136/bmjopen-2018-024355
9. Western B, Braga A, Hureau D, et al. Study retention as bias reduction in a hard-to-reach population. *Proceedings of the National Academy of Sciences* 2016;113(20):5477. doi: 10.1073/pnas.1604138113
10. Health Data Research UK. Health Data Research UK 2019 [Available from: www.hdruk.ac.uk.
11. CRIS. Clinical Records Interactive Search 2019 [Available from: https://www.maudsleybrc.nihr.ac.uk/facilities/clinical-record-interactive-search-cris/.
12. Farr Institute. The Farr Institute for Health Informatics Research 2019 [Available from: https://farrinstitute.org.
13. Gray L. The importance of post hoc approaches for overcoming non-response and attrition bias in population-sampled studies. *Social psychiatry and psychiatric epidemiology* 2016;51(1):155-57. doi: 10.1007/s00127-015-1153-8 [published Online First: 2015/11/28]
14. Norman JE, Morris C, Chalmers J. The effect of changing patterns of obstetric care in Scotland (1980-2004) on rates of preterm birth and its neonatal consequences: perinatal database study. *PLoS medicine* 2009;6(9):e1000153-e53. doi: 10.1371/journal.pmed.1000153 [published Online First: 2009/09/22]
15. Price A, Bryson H, Smith A, et al. Processes for engaging and retaining women who are experiencing adversity in longitudinal health services research. *BMC Health Serv Res* 2019;19(1):833-33. doi: 10.1186/s12913-019-4698-5

For peer review only - http://bmjopen.bmj.com/site/about/guidelines.xhtml

16. Aldhous MC, Bhatia R, Pollock R, et al. HPV infection and pre-term birth: a data-linkage study using Scottish Health Data. *Wellcome Open Res* 2019;4:48-48. doi: 10.12688/wellcomeopenres.15140.1
17. Hsieh DC, Smithers LG, Black M, et al. Implications of vaginal instrumental delivery for children's school achievement: A population-based linked administrative data study. *Australian and New Zealand Journal of Obstetrics and Gynaecology* 2019;59(5):677-83. doi: 10.1111/ajo.12952
18. Hann M, Roberts SA, D'Souza SW, et al. The growth of assisted reproductive treatment-conceived children from birth to 5 years: a national cohort study. *BMC Med* 2018;16(1):224-24. doi: 10.1186/s12916-018-1203-7
19. Martin P, Cortina-Borja M, Newburn M, et al. Timing of singleton births by onset of labour and mode of birth in NHS maternity units in England, 2005-2014: A study of linked birth registration, birth notification, and hospital episode data. *PloS one* 2018;13(6):e0198183-e83. doi: 10.1371/journal.pone.0198183
20. Thornton CE, Dahlen HG. Born before arrival in NSW, Australia (2000–2011): a linked population data study of incidence, location, associated factors and maternal and neonatal outcomes. *BMJ Open* 2018;8(3):e019328. doi: 10.1136/bmjopen-2017-019328
21. Harper G. Linkage of Maternity Hospital Episode Statistics data to birth registration and notification records for births in England 2005–2014: Quality assurance of linkage of routine data for singleton and multiple births. *BMJ Open* 2018;8(3):e017898. doi: 10.1136/bmjopen-2017-017898
22. Dattani N, Macfarlane A. Linkage of Maternity Hospital Episode Statistics data to birth registration and notification records for births in England 2005-2014: methods. A population-based birth cohort study. *BMJ open* 2018;8(2):e017897-e97. doi: 10.1136/bmjopen-2017-017897
23. Callander EJ, Fenwick J, Donnellan-Fernandez R, et al. Cost of maternity care to public hospitals: a first 1000-days perspective from Queensland. *Australian Health Review* 2019;43(5):556-64.
24. Howard LM, Piot P, Stein A. No health without perinatal mental health. *The Lancet* 2014;384(9956):1723-24. doi: 10.1016/S0140-6736(14)62040-7
25. Stein A, Pearson RM, Goodman SH, et al. Effects of perinatal mental disorders on the fetus and child. *The Lancet* 2014;384(9956):1800-19. doi: http://dx.doi.org/10.1016/S0140-6736(14)61277-0
26. Goldstein RF, Abell SK, Ranasinha S, et al. Association of Gestational Weight Gain With Maternal and Infant Outcomes: A Systematic Review and Meta-analysis. *JAMA* 2017;317(21):2207-25. doi: 10.1001/jama.2017.3635
27. Kahn RS, Zuckerman B, Bauchner H, et al. Women's Health After Pregnancy and Child Outcomes at Age 3 Years: A Prospective Cohort Study. *American Journal of Public Health* 2002;92(8):1312-18. doi: 10.2105/AJPH.92.8.1312
28. HRA. Health Research Authority. Section 251 and the Confidentiality Advisory Group (CAG) 2019 [Available from: http://www.hra.nhs.uk/about-the-hra/our-committees/section-251/ accessed 11 June 2019.
29. Stewart R, Soremekun M, Perera G, et al. The South London and Maudsley NHS Foundation Trust Biomedical Research Centre (SLAM BRC) case register: development and descriptive data. *BMC Psychiatry* 2009;9(1):51. doi: 10.1186/1471-244X-9-51

For peer review only

30. Fernandes AC, Cloete D, Broadbent MTM, et al. Development and evaluation of a de-identification procedure for a case register sourced from mental health electronic records. *BMC medical informatics and decision making* 2013;13:71-71. doi: 10.1186/1472-6947-13-71
31. Perera G, Broadbent M, Callard F, et al. Cohort profile of the South London and Maudsley NHS Foundation Trust Biomedical Research Centre (SLaM BRC) Case Register: current status and recent enhancement of an Electronic Mental Health Record-derived data resource. *BMJ Open* 2016;6(3):e008721. doi: 10.1136/bmjopen-2015-008721
32. Ristevski B, Chen M. Big Data Analytics in Medicine and Healthcare. *J Integr Bioinform* 2018;15(3):20170030. doi: 10.1515/jib-2017-0030
33. Zhang X, Pérez-Stable EJ, Bourne PE, et al. Big Data Science: Opportunities and Challenges to Address Minority Health and Health Disparities in the 21st Century. *Ethn Dis* 2017;27(2):95-106. doi: 10.18865/ed.27.2.95
34. CleverMed. BadgerNet Platform 2017 [Available from: https://www.clevermed.com/badgernet/badgernet-platform/.
35. NICE NIfH. Antenatal care for uncomplicated pregnancies 2008 [updated February 2019. Available from: https://www.nice.org.uk/guidance/cg62/chapter/1-Guidance2020.

For peer review only

For peer review only - http://bmjopen.bmj.com/site/about/guidelines.xhtml

Figure 1 - Data flow diagram for the eLIXIR Partnership linkage

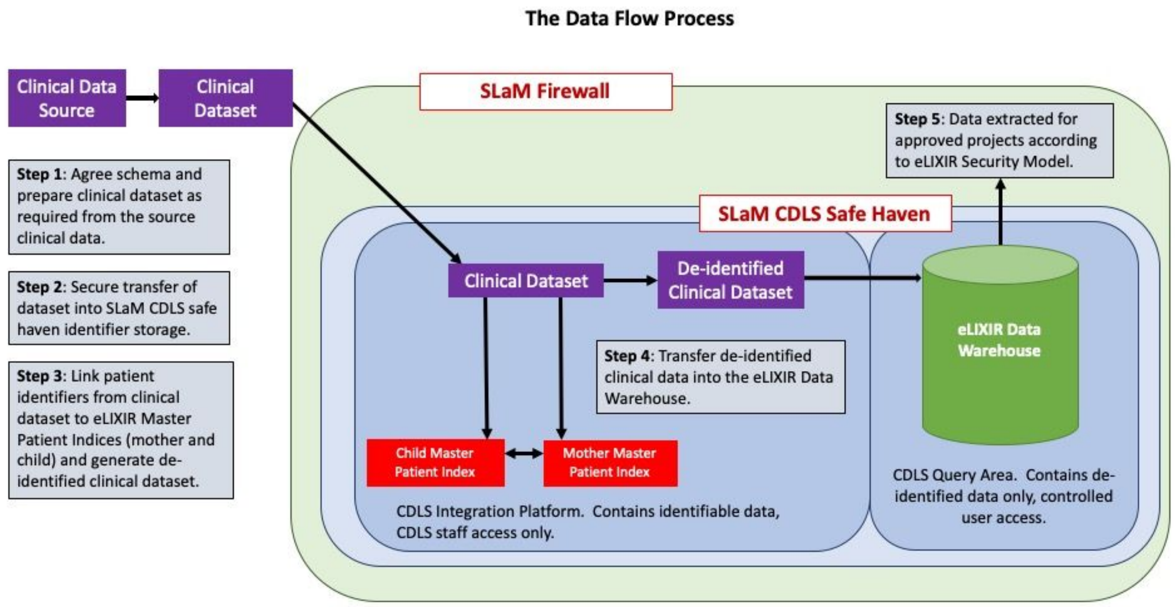

For peer review only

BMJ Open

Cohort Profile: The eLIXIR Partnership - a maternity-child data linkage for Life Course Research in South London, UK.

Journal:	*BMJ Open*
Manuscript ID	bmjopen-2020-039583.R1
Article Type:	Cohort profile
Date Submitted by the Author:	28-Jul-2020
Complete List of Authors:	Carson, Lauren; Institute of Psychiatry Psychology and Neuroscience, Psychological Medicine<br>Azmi, Borscha; Institute of Psychiatry Psychology and Neuroscience, Psychological Medicine<br>Jewell, Amelia; South London and Maudsley NHS Foundation Trust, NIHR Maudsley Biomedical Research Centre<br>Taylor, Clare; Women's College Hospital, Women’s College Research Institute<br>Flynn, Angela; King's College London, Women and Children's Health<br>Gill, Carolyn; King's College London, Women and Children's Health; Guy's and Saint Thomas' Hospitals NHS Trust, Women’s Health Academic Centre KHP<br>Broadbent, Matthew; South London and Maudsley NHS Foundation Trust, NIHR Maudsley Biomedical Research Centre<br>Howard, Louise; Institute of Psychiatry Psychology and Neuroscience, Section of Women's Health; South London and Maudsley NHS Foundation Trust, NIHR Maudsley Biomedical Research Centre<br>Stewart, Robert; Institute of Psychiatry Psychology and Neuroscience, Psychological Medicine; South London and Maudsley NHS Foundation Trust, NIHR Maudsley Biomedical Research Centre<br>Poston, Lucilla; King's College London, Department of Women and Children's Health
**Primary Subject Heading**:	Health informatics
Secondary Subject Heading:	Mental health, Obstetrics and gynaecology, Epidemiology
Keywords:	OBSTETRICS, EPIDEMIOLOGY, PSYCHIATRY, Health informatics < BIOTECHNOLOGY & BIOINFORMATICS

SCHOLARONE™
Manuscripts

I, the Submitting Author has the right to grant and does grant on behalf of all authors of the Work (as defined in the below author licence), an exclusive licence and/or a non-exclusive licence for contributions from authors who are: i) UK Crown employees; ii) where BMJ has agreed a CC-BY licence shall apply, and/or iii) in accordance with the terms applicable for US Federal Government officers or employees acting as part of their official duties; on a worldwide, perpetual, irrevocable, royalty-free basis to BMJ Publishing Group Ltd ("BMJ") its licensees and where the relevant Journal is co-owned by BMJ to the co-owners of the Journal, to publish the Work in this journal and any other BMJ products and to exploit all rights, as set out in our licence.

The Submitting Author accepts and understands that any supply made under these terms is made by BMJ to the Submitting Author unless you are acting as an employee on behalf of your employer or a postgraduate student of an affiliated institution which is paying any applicable article publishing charge ("APC") for Open Access articles. Where the Submitting Author wishes to make the Work available on an Open Access basis (and intends to pay the relevant APC), the terms of reuse of such Open Access shall be governed by a Creative Commons licence – details of these licences and which Creative Commons licence will apply to this Work are set out in our licence referred to above.

Other than as permitted in any relevant BMJ Author's Self Archiving Policies, I confirm this Work has not been accepted for publication elsewhere, is not being considered for publication elsewhere and does not duplicate material already published. I confirm all authors consent to publication of this Work and authorise the granting of this licence.

For peer review only

Cohort Profile: The eLIXIR Partnership - a maternity-child data linkage for Life Course Research in South London, UK.

Corresponding Author: Lauren E Carson, Department of Psychological Medicine, Institute of Psychiatry, Psychology and Neuroscience, King's College London, PO84, 16 De Crespigny Park, London, SE5 8AF, UK; lauren.carson@kcl.ac.uk

Borscha Azmi, Department of Psychological Medicine, Institute of Psychiatry, Psychology and Neuroscience, King's College London, UK

Amelia Jewell, NIHR Maudsley Biomedical Research Centre, South London and Maudsley NHS Foundation Trust, London, UK

Clare Taylor, Women's College Research Institute, Women's College Hospital, Toronto, Canada

Angela C Flynn, Department of Women and Children's Health, School of Life Course Sciences, King's College London, UK

Carolyn Gill, Department of Women and Children's Health, King's College London, UK and Women's Health Academic Centre KHP, Guy's and St Thomas' NHS Foundation Trust, UK

Matthew Broadbent, NIHR Maudsley Biomedical Research Centre, South London and Maudsley NHS Foundation Trust, London, UK

Louise M Howard, Section of Women's Mental Health, Institute of Psychiatry, Psychology and Neuroscience, King's College London, UK and NIHR Maudsley Biomedical Research Centre, South London and Maudsley NHS Foundation Trust, London,

Robert Stewart, Department of Psychological Medicine, Institute of Psychiatry, Psychology and Neuroscience, King's College London, UK and NIHR Maudsley Biomedical Research Centre, South London and Maudsley NHS Foundation Trust, London, UK

Lucilla Poston, Department of Women and Children's Health, School of Life Course Sciences, King's College London, UK

On behalf of the eLIXIR Partnership.

Word Count: 3,994

For peer review only

For peer review only - http://bmjopen.bmj.com/site/about/guidelines.xhtml

ABSTRACT

Purpose

Linked maternity, neonatal and maternal mental health records were created to support research into the early life origins of physical and mental health, in mothers and children. The Early Life Cross Linkage in Research (eLIXIR) Partnership was developed in 2018, generating a repository of real-time, de-identified, structured data derived from the electronic health record systems of two Acute and one Mental Health Care NHS Provider in South London. We present early descriptive data for the linkage database, and the robust data security and governance structures, and describe the intended expansion of the database from its original development. Additionally, we report details of the accompanying eLIXIR Research Tissue Bank of maternal and neonatal blood samples.

Participants

Descriptive data were generated from the eLIXIR database from 1 October 2018 to 30 June 2019. Over 17,000 electronic patient records were included.

Findings to date

10,207 women accessed antenatal care from the 2 NHS maternity services, with 8,405 deliveries (8,772 infants). This diverse, inner city maternity service population were born in over 170 countries with an ethnic profile of 46.1% White, 19.1% Black, 7.0% Asian, 4.1% Mixed and 4.1% other. Of the 10,207 women, 11.6% had a clinical record in mental health services with 3.0% being treated during their pregnancy. This first data extract included 947 infants treated in the neonatal intensive care unit (NICU), of whom 19.1% were postnatal transfers from external healthcare providers.

Future plans

Electronic health records provide potentially transformative information for life course research, integrating physical and mental health disorders and outcomes in routine clinical care. The eLIXIR database will grow by ~14,000 new maternity cases annually, in addition to providing child follow-up data. Additional datasets will supplement the current linkage from other local and national resources, including primary care and hospital inpatient data for mothers and their children.

For peer review only

STRENGTHS AND LIMITATIONS

- eLIXIR is a unique population-based database incorporating clinical data from maternity, neonatal and mental health records enabling life course studies of physical and mental health in a large, diverse, inner city, UK population.
- Studies undertaken using eLIXIR will have implications for local health care improvement but also potential to provide evidence to influence healthcare in similar national/global settings.
- Missingness and inaccuracy in all routine/administrative clinical databases will be a key limitation to this database.
- The representativeness of the cohort to the UK population is limited to mixed, inner-urban catchments.

For peer review only

INTRODUCTION

Investment in health in the earliest stages of life is increasingly recognised as a means to improve the life course of health; beginning *in utero*, through to infancy, childhood and into adulthood [1 2]. Much of the supporting evidence underpinning a 'life course approach' to disease prevention from pregnancy and infancy onwards has been accrued from large birth cohorts [3-6]; however, directly recruited cohorts are by definition drawn from individuals recruited over a pre-specified and limited time period and thereby become rapidly outdated as temporal shifts in population demography, lifestyle and ethnicity occur. Moreover, facility-centred follow-up is expensive and difficult to sustain [7 8]. Sample attrition is common and can introduce significant methodological biases that may affect the validity of investigations into novel risk outcomes [9]. In addition, direct recruitment may result in cohorts with limited representation of the target population because of selective inclusion. Population-based registries also offer insight into rarer diseases and outcomes, not feasible in current research cohorts. Linked administrative data are increasingly used to provide evidence to guide policy and clinical management [10-12]. The longitudinal nature of such case registers, their size, and coverage of defined populations provide an increasingly attractive alternative to the study of birth cohorts for defining the early life exposures that contribute to the population burden of physical and mental health disorders. These can provide longitudinal information on large numbers of women and children, as well as the potential for linkage with a widening portfolio of available local and national datasets to follow health from birth to adulthood (https://digital.nhs.uk/data-and-information/data-collections-and-data-sets/data-sets). Both inclusion and attrition bias of traditional birth cohorts can be overcome through routine comprehensive health records, as these can capture rich clinical data in a given population on all women receiving antenatal care, and their infants [13]. Whilst well established in Scandinavian countries, national birth registries in the UK have not been widely used in linkage programmes using infant and childhood data, although population registry data from Scotland have for many years provided information on relationships between maternal and neonatal outcomes which has informed clinical guidelines in the UK and beyond [14]. Several linkages of clinical maternity and infant data have nonetheless shown the feasibility of the approach, and usefulness, for example in aligning hospital maternity data with national birth registration datasets, or birth registration datasets with Hospital Episode Statistics , or using UK primary care pregnancy data to create a pregnancy register [15-25].

It is well established that maternal physical and mental wellbeing in pregnancy and the postpartum period can strongly influence neonatal outcome and the physical and mental health of the child [26-29]. To our knowledge, no clinical data linkages in maternity or neonatal services have to date

For peer review only

incorporated clinical information from maternity, neonatal and mental health services into a single continuum to interrogate these associations at a population level. The Early Life Cross Linkage in Research (eLIXIR) Partnership has been developed to address these relationships from early pregnancy, the perinatal period and beyond into later life. Funded by the Medical Research Council (MRC) in 2017, the partnership is a multidisciplinary academic collaboration which aims to combine maternal, infant and child health data into a single resource to allow information from large numbers of mothers, babies and children to be investigated over an unlimited time period. The intention is to provide a naturally accumulating database to support investigations into associations between physical and mental health in mother and child.

The eLIXIR Partnership provides a mechanism through which research datasets can be linked to clinical records, under appropriate and approved levels of anonymity and data security. An added benefit is the potential to incorporate data from multiple sources, e.g. health, environment, social, and education. There are, however, important ethical and legal considerations, as well as technical security requirements, if linkages are to be performed between sources of routinely collected clinical data, and exemption from individual consent to be permissible [30].

Another aspect of the eLIXIR Partnership is the eLIXIR Research Tissue Bank established to link the routinely collected maternal and neonatal clinical data with biological samples. This has an advantage over static cohort studies by providing a "dynamic" collection of samples, enabling identification of population trends, and influences of new clinical interventions. The provision of samples from women attending antenatal care will provide a unique biobank to address mechanisms of common and rarer complications in pregnancy and in neonatal life, and their consequences for the longer-term health of the mother and child. Common complications will include gestational diabetes, mental illness, prematurity and pre-eclampsia. Similarly, by the provision of samples from neonatal intensive care, eLIXIR will contribute to a better understanding of neonatal morbidity and mortality.

With records of over 14,000 individual births per year, eLIXIR has the potential to become one of the largest mother-infant-child datasets in Europe. This has been facilitated by King's Health Partners (KHP): an Academic Health Sciences Centre which brings together one academic institution (King's College London (KCL)) and three NHS Foundation Trusts (Guys and St Thomas' NHS Foundation Trust (GSTT), King's College Hospital NHS Foundation Trust (KCH) and South London and Maudsley NHS Foundation Trust (SLaM)).

For peer review only - http://bmjopen.bmj.com/site/about/guidelines.xhtml

This manuscript details the technical and procedural elements in place to safeguard the legal and ethical rights of service users during the development and use of the eLIXIR database, and to present the demographic profile of the eLIXIR population. Both technical and procedural elements draw strongly on experience gained in setting up the Clinical Record Interactive Search (CRIS) data resource at the Maudsley National Institute for Health Research (NIHR) Biomedical Research Centre (BRC) [31-33].

Benefits of the system

Large data linkage platforms, such as that created by the eLIXIR Partnership, provide a unique data warehouse through which important epidemiological questions can be asked – in the case of eLIXIR, within a large and diverse inner-city population. The ability to conduct these linkages allows not only the collection of a wide range of longitudinal health and social data, but also the capacity to support life course data analysis. The potential benefits arising from the use of clinical record 'big data' have been widely reported, and research databases such as eLIXIR are likely to increase in number due to the powerful and cost-effective nature of this research method [34 35]. eLIXIR is one of the first longitudinal research databases which from early pregnancy onwards using routinely collected clinical data from maternity, neonatal and mental health services that does not rely on a recruited cohort of participants.

COHORT DESCRIPTION

Data sources

Maternity and neonatal data was obtained from GSTT and KCH, and mental health data from SLaM. GSTT provide a full range of hospital and community services for people in Lambeth, Southwark and Lewisham, as well as specialist care for patients from further afield including referrals for high risk pregnancies and neonatal complications. Similarly, KCH serves the boroughs of Lambeth, Southwark, and Lewisham, but also Bromley, with specialist services to patients across a wider catchment area, including referrals for obstetrics and fetal medicine. SLaM provides comprehensive mental health services to a geographic catchment of over 1.2 million residents in four south London boroughs, Croydon, Lambeth, Lewisham and Southwark, as well as some regional/national specialist mental health services.

Maternity, birth and neonatal intensive care data

The BadgerNet Platform (CleverMed) for routine clinical data is used extensively across the UK to create electronic patient records that capture early pregnancy community based events and hospital

For peer review only - http://bmjopen.bmj.com/site/about/guidelines.xhtml

based events for low and high risk pathways of care (BadgerNet Maternity), and neonatal intensive care, neonatal transport, paediatric intensive care, neurology referrals and adult intensive care data (BadgerNet Neonatal). Within GSTT and KCH the BadgerNet platforms are used for recording maternal/infant personal data, demographics, clinical history, clinic data (maternity only) and hospital episode data. The BadgerNet system records clinical records on a Single Care Record system which is nationally hosted [36]. Although feasible within the BadgerNet system, linkage between maternity and neonatal data is not routinely conducted.

Mental health data

Clinical records have been fully electronic across all SLaM NHS Trust mental health services since April 2006, using the bespoke electronic Patient Journey System (ePJS) which incorporated legacy data from earlier service specific electronic health records (EHRs). The Clinical Record Interactive Service (CRIS) platform[33] was developed in 2007–2008 and consists of a series of data-processing pipelines which both structure and de-identify PJS fields, rendering de-identified data from the full clinical record available at the researcher interface, with search and database assembly functionality facilitated by a front end, designed for non-technical use. The de-identifying process and its effectiveness, including the masking of identifying information in open-text fields and the generation of a pseudonymised identifier (CRIS ID), have been previously described [32]. The wider patient-led oversight and security models for CRIS have not changed significantly since it was established [31-33]. Ethical approval was obtained for CRIS as a de-identified database for secondary analysis (Oxford C Research Ethics Committee, reference 18/SC/0372). In terms of cohort coverage, all SLaM care (including diagnoses, medication and services provided), is represented on CRIS, including Improving Access to Psychological Therapies data (IAPT; a large primary care service providing short term psychological therapies).

Data linkage hosting environment

Data for eLIXIR are managed and stored at the Clinical Data Linkage Service (CDLS) at SLaM: an impartial trusted third-party service that provides researchers access to linked clinical data in accordance with the strict governance conditions and processes agreed with relevant data controllers. The CDLS is managed by a small, dedicated team of informaticians, IT, and Information Governance (IG) professionals (https://www.maudsleybrc.nihr.ac.uk/facilities/clinical-record-interactive-search-cris/), and currently hosts a range of datasets already linked with the SLaM CRIS mental health case register (e.g. Hospital Episode Statistics, National Cancer Registry, ONS death certification, Lambeth DataNet primary care records, and National Pupil Database). The backbone of

For peer review only - http://bmjopen.bmj.com/site/about/guidelines.xhtml

the eLIXIR database consists of a 'master patient index' (Figure 1) allowing data to be robustly linked within an appropriately secure environment according to data specifications.

The CDLS hosts both source data and the master patient index, on behalf of the eLIXIR Partnership, on a secure server within the NHS firewall with role-restricted access. The CDLS additionally provides a data extraction service which meets security requirements, creating bespoke datasets for approved research use. Such derived data are managed by the approved research team and are hosted at all times on a dedicated drive within the NHS for analysis in this domain using hosted software already available at SLaM.

Four distinct services are offered by the CDLS as the data processor for the eLIXIR data. First, CDLS provides advice on permissions, approvals and contracts. These include consideration of academic, technical, legal and ethical requirements. Second, CDLS facilitates data linkages either within the CDLS safe haven or via a third party, coordinating the secure transfer of data. Third, the CDLS is responsible for the secure storage of linked data in accordance with predefined information governance and security standards. Fourth, the CDLS, as the custodian for the linked data, prepares and extracts bespoke and prespecified databases for approved eLIXIR projects and provides these to researchers. Therefore, there is no direct access by researchers to the full linked data files, enhancing data protection and confidentiality.

Data linkage procedures and resources

The eLIXIR Partnership uses common identifiers (e.g. hospital number, NHS number, name, and date of birth) to link between maternity, neonatal and mental health clinical data, which is undertaken by CDLS staff, not researchers. Matching is undertaken using deterministic matching techniques on a given set of identifiers. Two records are said to match if all or some of the identifiers are identical, as defined by a hierarchical set of match-ranks. This creates a single master patient index including patient anonyms (mother and child), binary variables for presence/absence on each data source, and key demographics. The source datasets and master patient index are stored by the CDLS behind the NHS firewall and linked data are extracted on a project-by-project basis by a CDLS informatician containing the variables and samples necessary for each study, with a study-specific encrypted anonym which contains completely de-identified data. In this way databases are not stored in linked format. The clinical data from maternity and neonatal services are extracted from BadgerNet systems at both GSTT and KCH by ICT staff at each site. These data are then sent securely to the staff at the SLaM CDLS, and linked, using their common identifiers, with data from the CRIS system to

For peer review only - http://bmjopen.bmj.com/site/about/guidelines.xhtml

incorporate mental health clinical data, where present, for each patient. This results in a comprehensive data resource to which researchers can apply for extracted data across maternity, neonatal and/or mental health services. Figure 1 details the dataflow for the eLIXIR Partnership database. Match quality is assured as 100% of infants born within the eLIXIR dataset were matched with their mothers' records with the BadgerNet System.

Information governance framework

Results from linkages, current and prior, are stored within the CDLS safe haven, and the CDLS plays a key role in wider governance, supplementing the role of eLIXIR-specific oversight and data security, including the secure handling and storage of identifier fields required for data linkage. Section 251 (s251) of the NHS Act 2006 allows the common law duty of confidentiality to be set aside in specific circumstances where anonymised information is not sufficient and where 'opt-in' patient consent is not practical. Opt-out information and details of the project are given to each patient entering maternity and neonatal services, and patients have the option of opting out of the programme at any time. Approval under this legal framework was granted by the Health Research Authority (HRA) Confidentiality Advisory Group (CAG) to the eLIXIR team for all the above linkages, which allow data to be available in an identifiable format to a small number of data processing staff in accordance with data sharing contracts between the data provider institutions (HRA CAG Ref: 18/CAG/0040). Therefore, for current and future data linkages within eLIXIR, ethical (REC) and s251 approval is required either through amendments to our existing agreements or new applications to these regulatory bodies. Activity for projects using linked data sets held by eLIXIR is audited by the eLIXIR Oversight Committee, helping to ensure that the researcher's project requirements (e.g. clinical research, surveillance, service improvement or audit) are met, and that projects progress within the agreed policy and practice framework. The primary role of the Committee is to provide the operational management of eLIXIR as identified in the eLIXIR Security Model and protocol for the eLIXIR Research Tissue Bank. In so doing, the Committee seeks to promote the scientific and ethical principles that should govern the use of eLIXIR data and seeks to represent stakeholders [who include the KHP Caldicott Guardians (individuals responsible for research governance at each NHS Trust site), service users, clinical professionals, lay persons, and academics] and reflect their views and interests.

Public and patient involvement

Public and patient involvement (PPI) involvement was incorporated throughout the development of the eLIXIR Partnership. The concept of the eLIXIR Partnership was presented to a variety of PPI

For peer review only - http://bmjopen.bmj.com/site/about/guidelines.xhtml

groups, including the Maudsley Biomedical Research Centre (BRC) Data Linkage Service User and Carer Advisory Group[37], Lambeth HealthWatch and the Young Persons Advisory Group at Great Ormond Street Hospital. PPI is ensured in the decision-making process of approving eLIXIR projects through lay member representation on the eLIXIR Oversight Committee. The eLIXIR Oversight Committee reviews and approves all projects using eLIXIR data.

Research Tissue Bank

The eLIXIR Research Tissue Bank is a prospective biobank of samples from pregnant women and infants being treated by GSTT. The Research Tissue Bank is integrated into the King's College London HTA governance structure with active recruitment and collection of samples. All pregnant women aged over 16 years who are willing and able to give informed consent, and infants admitted to the neonatal unit that have parental consent, are eligible for inclusion in the eLIXIR Research Tissue Bank (Cambridge East Research Ethics Committee, reference 18/EE/0120).

Maternal blood sample collection

Eligible women are recruited at the time of routine antenatal care venepuncture (11-15 weeks' gestation or later transfer of care or later antenatal care attendance) at GSTT. Women who agree to participate give written informed consent and an extra blood sample is collected at the same time as routine venepuncture, maximum volume 12ml (2 x 6ml tubes).

Infant blood sample collection

It is our intention also to recruit samples from infants admitted to the neonatal intensive care unit (NICU), when blood is drawn for routine tests. Following written informed parental consent, residual blood from routine samples, which otherwise would be discarded, will be retained and collected. The samples will be processed and stored in a similar manner to the maternal blood samples. Following birth in the community setting (home visits), every infant, whose mother has provided consent, is offered new-born blood spot screening to exclude metabolic disorders. A health professional pricks the baby's heel to collect 4 drops of blood on a card. For the biobank, an extra card to those taken for clinical purposes is used to collect extra bloodspots from the infant, after the routine spots are collected using the same heel prick. This sample is posted back to the eLIXIR team and stored in the research laboratory prior to transfer to a central storage facility.

All samples are transferred for processing in the research laboratory. After processing, all tubes are labelled with a study specific barcode and entered on to a study specific database (FreezerPro©).

For peer review only - http://bmjopen.bmj.com/site/about/guidelines.xhtml

The samples are stored in -80°C freezers short-term, before being transported to a central storage facility (NIHR BioResource, Milton Keynes).

FINDINGS TO DATE

Maternal, Birth and Birth Outcomes

From the first data extraction (1 October 2018 - 30 June 2019) 10,207 women accessed antenatal care through GSTT or KCH maternity services with 8,405 deliveries (8,772 infants). This diverse, inner city antenatal population were born in over 170 countries with a heterogeneous distribution across ethnic groups (Table 1). Most were born outside the UK but most reported English as their primary language. Women were booked on average at 11.6 weeks gestation, which is slightly higher than the national guidelines of before 10 weeks [38]. The most common physical conditions experienced were; gynaecological problems (14%), asthma (8%) and pre-existing diabetes (6%). In addition, around one in five reported mental health problems, 3% were recorded as being exposed to female genital mutilation and 4% reported being current smokers at the time of their antenatal appointment (Table 1).

Table 1 – Characteristics of women attending antenatal booking appointments at Guy's & St. Thomas' NHS Foundation Trust (GSTT) and King's College Hospital NHS Foundation Trust (KCH) between 1st October 2018 and 30th June 2019.

	GSTT (n=5,700)	*KCH (n=4,507)*	*Across both NHS Trusts (n=10,207)*
Age of mother at booking in years (mean ± SD)	32.7±5.5	32.7±5.6	32.7±5.5
Ethnicity N (%)			
White	2,560 (44.9%)	2,151 (47.7%)	4,711 (46.1%)
Black	862 (15.0%)	1,089 (24.3%)	1,951 (19.1%)
Asian	474 (8.3%)	248 (5.6%)	722 (7.0%)
Mixed	218 (3.8%)	192 (4.3%)	410 (4.1%)
Other	248 (4.4%)	253 (5.6%)	501 (4.9%)
Not Recorded	1,338 (23.4%)	574 (12.7%)	1,912 (18.8%)
Born in the UK (%)	2,150 (37.7%)	2,358 (52.3%)	4,508 (44.1%)
Primary Language English (%).	3,711 (65.1%)	3,474 (77.1%)	7,185 (70.4%)
Gestational age at booking (weeks) (mean ± SD)	11.9±6.4	11.2±6.4	11.6±6.5
Parity (Mean ± SD)	0.7 ±1.1	0.8±1.1	0.8±1.1
Gravida (Mean ± SD)	2.2±1.5	2.4±1.6	2.3±1.6

For peer review only - http://bmjopen.bmj.com/site/about/guidelines.xhtml

Prior medical conditions			
Haematological disorder	231 (4.1%)	407 (9.0%)	638 (6.3%)
Thrombosis	57 (1.0%)	35 (0.8%)	90 (0.9%)
Cardiac disorders	103 (1.8%)	103 (2.3%)	206 (2.0%)
Hypertension	108 (2.0%)	85(1.9%)	193 (1.9%)
Renal disorders	155 (2.8%)	135 (3.0%)	290 (2.9%)
Asthma	401 (7.0%)	413 (9.3%)	814 (8.0%)
Lung disorders	14 (0.2%)	10 (0.3%)	24 (0.2%)
Diabetes	375 (6.6%)	257 (5.7%)	627 (6.2%)
Endocrine disorders	307 (5.4%)	205 (4.6%)	512 (5.1%)
Autoimmune disorders	83 (1.4%)	62 (1.4%)	145 (1.4%)
Epilepsy	43 (0.7%)	37 (0.8%)	80 (0.8%)
Neurological disorders	146 (2.6%)	355 (7.9%)	501 (5.0%)
Gastrointestinal disorders	265 (4.6%)	282 (6.2%)	547 (5.4%)
Gynaecological disorders	742 (13.0%)	673 (15.0%)	1415 (14.1%)
Liver disorders	46 (0.9%)	55 (1.2%)	101 (1.0%)
Bone disorders	38 (0.7%)	59 (1.4%)	97 (0.9%)
Joint disorders	98 (1.7%)	77 (1.7%)	175 (1.8%)
Back problems	173 (3.0%)	375 (8.4%)	548 (5.1%)
Genetic disorders	‡ND	‡ND	‡ND
Congenital abnormalities	29 (0.5%)	27 (0.7%)	56 (0.5%)
Other	85 (1.5%)	121 (2.6%)	206 (2.0%)
Mental health problems (current or historical; self-reported)	988 (13.8%)	1,147 (25.5%)	2,135 (20.9%)
Victim of Female Genital Mutilation (FGM) (%yes)	209 (3.7%)	137 (3.0%)	346 (3.4%)
Smoker at booking (%yes)	195 (3.4%)	199 (4.4%)	394 (3.9%)

‡Not Disclosed (ND). Actual numbers suppressed to reduce risk of statistical disclosure

With regard to birth episodes, twice as many women gave birth at GSTT than KCH with a mean gestational age at delivery of 38.8 weeks, and with 8% born prematurely (<37 completed weeks of gestation) and 3% born very prematurely (<34 weeks' gestation). Of the 8,405 births, 8,051 were singletons, 341 were twin births and 13 were triplets. Around half of deliveries were spontaneous

For peer review only - http://bmjopen.bmj.com/site/about/guidelines.xhtml

cephalic, around one in five were emergency (or unspecified) caesarean section, and 15% elective caesarean section. Rates of stillbirth and neonatal deaths were 0.6% and 0.4% respectively. The mean birthweight was 3257g with 17% small for gestational age (SGA) and 7% large for gestational age (LGA) (Table 2).

Table 2 – Characteristics of births and birth outcomes at Guy's & St. Thomas' NHS Foundation Trust (GSTT) and King's College Hospital NHS Foundation Trust (KCH) between 1st October 2018 and 30th June 2019.

	GSTT (n=5,697)	*KCH (n=2,708)*	*Across both NHS Trusts (n=8,405)*
Gestation at delivery (weeks)	38.8±2.4	38.9±2.3	38.8±2.4
Very premature (<34 weeks)	193 (3.4%)	91 (3.4%)	284 (3.4%)
Premature (>34 weeks - <37 weeks)	295 (5.2%)	117 (4.3%)	412 (4.9%)
Term (>37 weeks)	5209 (91.4%)	2708 (92.3%)	7709 (91.7%)
Number of infants born (number of pregnancies)			
Singleton	5459 (95.8%)	2592 (95.7%)	8051 (95.8%)
Twins	227 (4.0%)	114 (4.2%)	341 (4.1%)
Triplets	‡ND	‡ND	13 (0.2%)
Type of delivery			
Breech	32 (0.6%)	16 (0.6%)	48 (0.6%)
Elective Caesarean Section	862 (15.1%)	412 (15.2%)	1274 (15.2%)
Emergency or unspecified Caesarean Section	1132 (19.9%)	461 (17.0%)	1593 (19.0%)
Forceps	462 (8.1%)	180 (6.6%)	642 (7.6%)
Spontaneous Cephalic	2838 (49.8%)	1378 (50.9%)	4216 (50.2%)
Ventouse	371 (6.5%)	257 (9.5%)	628 (7.5%)
Birth outcome			
Live birth	5644 (99.1%)	2680 (99.0%)	8324 (99.0%)
Stillbirth or neonatal death	53 (0.9%)	28 (1.0%)	81 (1.0%)
Birth weight (grams)	3249.1±621.7	3273.3±621.25	3256.9±621.6
Small for Gestational Age (Birth Centile ≤10th)	551 (9.7%)	259 (9.6%)	810 (9.7%)
Large for Gestational Age (Birth Centile≥90th)	551 (6.6%)	172 (6.4%)	549 (6.6%)

‡Not Disclosed (ND). Actual numbers suppressed to reduce risk of statistical disclosure

For peer review only - http://bmjopen.bmj.com/site/about/guidelines.xhtml

Neonatal Intensive Care Unit admissions

Of the 947 infants that had been treated in neonatal intensive care units across both GSTT and KCH, 19.1% were postnatal transfers from an external Trust and 8.7% were in-utero transfers. The main reason for admission was for respiratory disease (28%) followed by preterm birth (22%). The average length of time spent in NICU was 15.8 days. Of outcomes following admission 46% were re-admitted to a postnatal ward, 29% were discharged home, and neonatal death occurred in 4%, the remainder being unknown from the data available (Table 3).

Table 3 - Characteristics of patients admitted to neonatal intensive care unit (NICU) at Guy's & St. Thomas' NHS Foundation Trust (GSTT) and King's College Hospital NHS Foundation Trust (KCH) between 1st October 2018 and 30th June 2019.

	GSTT (n=645)	*KCH (n=302)*	*Total (n=947)*
Gestational age (weeks)	35.2 (±5.0)	35.7 (±4.6)	35.3 (±4.9)
Birthweight (grams)	2541.1 (±1075.1)	2586.4 (±1022.2)	2555.6 (±1051.32)
Number of days on NNU	15.3±24.7	16.9(±26.2)	15.8 (±25.2)
Number of episodes of care			
1	476 (73.8%)	254 (84.1%)	730 (77.1%)
2	135 (20.9%)	39 (12.9%)	174 (18.4%)
3	‡ND	‡ND	30 (3.2%)
4+	‡ND	‡ND	13 (1.2%)
Readmission (yes)	25 (3.9%)	11 (3.6%)	36 (3.8%)
Consanguineous parents	‡ND	‡ND	11 (1.2%)
Reason for admission			
Preterm Birth	132 (20.5%)	73 (24.2%)	205 (21.6%)
Respiratory disease	172 (26.7%)	90 (29.8%)	262 (27.6%)
Cardiovascular disease	‡ND	‡ND	93 (9.8%)
Infection	11 (1.7%)	12 (4.0%)	23 (2.4%)
Jaundice	29 (4.5%)	11 (3.6%)	40 (4.2%)
Poor Feeding/Weight loss	‡ND	‡ND	21 (2.2%)
Gastro-intestinal disease	‡ND	‡ND	24 (2.5%)
Hypoglycaemia	34 (5.3%)	13 (4.3%)	47 (5.0%)

For peer review only - http://bmjopen.bmj.com/site/about/guidelines.xhtml

Convulsions or neurological disease	‡ND	‡ND	12 (1.3%)
Congenital abnormality	‡ND	‡ND	28 (2.3%)
Surgery	67 (10.4%)	27 (8.9%)	94 (9.9%)
Investigation/monitoring	26 (4.0%)	14 (4.6%)	40 (4.3%)
Poor condition at birth	‡ND	‡ND	22 (2.3%)
Other	19 (2.9%)	17 (5.6%)	36 (3.8%)
Maternal drug use	‡ND	‡ND	13 (1.4%)
Maternal smoking (n)	23 (3.6%)	20 (6.6%)	43 (4.5%)
Neonatal outcome			
Ward	281 (43.6%)	152 (50.3%)	433 (45.7%)
Home	181 (28.1%)	97 (32.1%)	278 (29.4%)
Died	27 (4.2%)	13 (4.3%)	40 (4.2%)
Unknown	156 (24.2%)	40 (13.2%)	196 (20.7%)

‡Not Disclosed (ND). Actual numbers suppressed to reduce risk of statistical disclosure

Mental Health

Of the 10,207 women attending antenatal care as registered in the eLIXIR database, 1,184 had a clinical record in secondary mental health services (SLaM) with 307 women actively being treated at the time of their pregnancy (201 under the care of Improving Access to Psychological Therapies (IAPT)) (Table4).

Table 4 - Mental health data (South London and Maudsley NHS Foundation Trust, SLaM) of women receiving antenatal care at Guy's & St. Thomas' NHS Foundation Trust (GSTT) and King's College Hospital NHS Foundation Trust (KCH) between 1st October 2018 and 30th June 2019.

	GSTT (n=5700)	*KCH (n=4507)*	*Across both NHS Trusts (n=10,207)*
Ever received treatment from SLaM	658 (11.5%)	526 (11.7%)	1184 (11.6%)
Under SLaM Care during pregnancy	180 (3.2%)	127 (2.8%)	307 (3.0%)
Received psychological therapy (through IAPT) in pregnancy from SLaM	113 (2.0%)	88 (2.0%)	201 (2.0%)

Research Tissue Bank

For peer review only - http://bmjopen.bmj.com/site/about/guidelines.xhtml

Following all necessary governance agreements samples were collected over a period of 3 months. A total of 1271 aliquoted samples (including EDTA, serum and whole blood) from 123 women were stored in the FreezerPro© system. In this period, 63.4% of women approached gave consent to take part.

STRENGTHS AND LIMITATIONS

The eLIXIR Partnership has developed a unique population-based database incorporating clinical data from maternity, neonatal and mental health records. This is supplemented by the eLIXIR Research Tissue Bank. Together this resource will provide the basis for additional linkages to enable life course studies of physical and mental health in a large, diverse, inner city, UK population. This will not only have implications for local health care improvement but also has the potential to provide evidence to influence healthcare in similar national/global settings.

A limitation common to all routine/administrative clinical databases is source data missingness and inaccuracy. This, in contrast, is an advantage of prospective research cohorts. Nonetheless eLIXIR provides opportunity for continuous feedback to the clinical provider on data absence or error. With regular meetings we already appraise the Trust IT teams of, for example, duplicate patient data entry and missing data, especially that required for the national Maternity Services Data Set (MSDS). Thus, eLIXIR and other similar research datasets can contribute directly to improved clinical reporting, and hence to better clinical care.

Another potential limitation is loss of data from women who move outside of the catchment area within the index pregnancy and thereafter, although it is our intention to supplement the linkages with data from national Hospital Episode Statistics (HES), to provide information on hospitalised outcomes. As with all administrative clinical datasets, research will be limited to that which can be conducted using information routinely collected in clinical care. The intended incorporation of research datasets offsets this to some extent. Additionally, clinical data entry may involve substantial human error, and data may be absent from the clinical records (i.e. missing). The representativeness of the cohort to the UK population is limited to mixed, inner-urban catchments. In addition, all three NHS Trusts involved in the eLIXIR Partnership incorporate specialisms or local expertise attracting national-level referrals. As a result, data may be skewed to patients with more severe or complex health issues.

There are several advantages, but also disadvantages to using de-identified electronic cohorts versus more traditional consented cohorts (e.g. Avon Longitudinal Study of Parents and Children (ALSPAC) or Born in Bradford (BiB)). The greatest advantage lies in the contemporary reporting of a population

For peer review only

compared with historical cohorts, and we are aware that Born in Bradford has embarked on a mother-child linkage involving all mothers who consent to provide their de-identified routine data, and that of their infants (e.g. Born in Bradford 2019; borninbradford.nhs.uk/what-we-do/pregnancy-early-years/born-in-bradford). This and other planned 'local' UK linkages provide opportunity for collaboration through meta-analysis to compare and contrast with other diverse UK populations. Another key advantage of de-identified records is the increased representativeness of underrepresented groups of patients, often missing from traditional cohorts. Also, this approach avoids the cost implications of consent and cohort maintenance. However, traditional cohorts have the advantage of much greater depth of biological and psychological information derived from procedures and validated questionnaires.

Plans for the future

As the eLIXIR database develops, expansions will incorporate health and social care data. The next phase of linkage will comprise a local primary care data resource, Lambeth DataNet (https://selondonccg.nhs.uk/in-your-area/lambeth/our-local-plans/) for which all necessary approvals are in place. Beyond-catchment hospitalisation data could be usefully captured, by linkage to national data sources, e.g. HES, as mentioned, which has been incorporated in the CRIS platform[33]. Subject to approval, later linkages will incorporate prescribing and education data, in addition to a broader range of local healthcare information as eLIXIR infants enter age ranges covered by other specialties. Finally, replication of this data-linkage model in other geographical settings in the UK would offer the potential to develop a national data network allowing both larger research cohorts and cross-site replication.

COLLABORATION

We have established a research database of maternity, neonatal and mental health clinical data not only combining maternal physical and mental health clinical data during pregnancy and later neonatal health, but also provides added value through potential for addition of biological measurements (i.e. omics data) from the eLIXIR Tissue Bank samples. The eLIXIR Programme has the capacity to continue to grow and develop exponentially, through internal and external collaborations, with small levels of attrition to follow-up and the ability to be used for both common and rare research outcomes. Furthermore, unlike comparable research programmes, the population we sample from is diverse on both an ethnicity and sociodemographic level providing a richness of data which has the potential to lead to exciting research findings.

For peer review only

FURTHER DETAILS

Data sharing statement

Researchers can apply for data access and biomaterial by submitting a research application form to the eLIXIR team. The eLIXIR website provides information on the application process (http://www.guysandstthomasbrc.nihr.ac.uk/microsites/elixir). To apply to use data from eLIXIR, researchers must complete a Research Application Form (RAF), available on the study website, and submit this, via email, to the eLIXIR Oversight Committee for their consideration and approval. The associated costs with accessing data are study dependent. Basic infrastructure for data storage and CDLS services is provided by the core team. Individual project costs are determined by the length of study and which datasets are required. Costs to the researcher include data access (via VPN), data cleaning and statistical support. The eLIXIR Partnership provides the infrastructure for data linkage, but external funding will be sought for additional linkage to external datasets.

Funding declaration

This work was supported by an MRC Partnership Grant [MR/P003060/1].

LC and LH receives salary support from the National Institute for Health Research (NIHR) Applied Research Collaboration South London (NIHR ARC South London) at King's College Hospital NHS Foundation Trust. LC was additionally supported by a Medical Research Council Mental Health Data Pathfinder Award to King's College London. LC, AJ, MB, LH, RS are part-funded by the National Institute for Health Research (NIHR) Biomedical Research Centre at the South London and Maudsley NHS Foundation Trust and King's College London. AF and LP are supported by Tommy's Charity and King's College London. CG and LP are supported by the National Institute for Health Research (NIHR) Biomedical Research Centre at Guy's and St. Thomas' NHS Foundation Trust.

Conflict of interest

RS declares research support received in the last 5 years from Roche, Janssen, GSK and Takeda.

Disclaimer

The views expressed are those of the authors and not necessarily those of the NHS, the NIHR, the MRC or the Department of Health.

Ethics approval

For peer review only

Oxford C REC (18/SC/0086) and Cambridge East REC (18/EE/0120).

Contributorship statement

LC, LH, RS, LP contributed to the conception of this article. LC, BA, AJ, CT, AF, CG, LH, RS, LP were involved in manuscript writing and revision. LC, MB, LP were involved in data analysis and interpretation. All authors read and approved the final manuscript.

For peer review only

References

1. Mikkelsen B, Williams J, Rakovac I, et al. Life course approach to prevention and control of non-communicable diseases. *Bmj* 2019;364:l257. doi: 10.1136/bmj.l257
2. Kuruvilla S, Sadana R, Montesinos EV, et al. A life-course approach to health: synergy with sustainable development goals. *Bull World Health Organ* 2018;96(1):42-50. doi: 10.2471/BLT.17.198358 [published Online First: 2017/11/23]
3. Wright J, Small N, Raynor P, et al. Cohort Profile: The Born in Bradford multi-ethnic family cohort study. *International journal of epidemiology* 2012;42(4):978-91. doi: 10.1093/ije/dys112
4. Sebert S, Lowry E, Aumüller N, et al. Cohort Profile: The DynaHEALTH consortium – a European consortium for a life-course bio-psychosocial model of healthy ageing of glucose homeostasis. *International journal of epidemiology* 2019;48(4):1051-51k. doi: 10.1093/ije/dyz056
5. Boyd A, Golding J, Macleod J, et al. Cohort Profile: the 'children of the 90s'--the index offspring of the Avon Longitudinal Study of Parents and Children. *International journal of epidemiology* 2013;42(1):111-27. doi: 10.1093/ije/dys064
6. Scholtens S, Smidt N, Swertz MA, et al. Cohort Profile: LifeLines, a three-generation cohort study and biobank. *International journal of epidemiology* 2014;44(4):1172-80. doi: 10.1093/ije/dyu229
7. Overy CR, LA; Tansey, EM, ed. History of the Avon Longitudinal Study of Parents and Children , C 1980-2000. History of the Avon Longitudinal Study of Parents and Children, C 1980-2000; 2011. Queen Mary, University of London.
8. Downs JM, Ford T, Stewart R, et al. An approach to linking education, social care and electronic health records for children and young people in South London: a linkage study of child and adolescent mental health service data. *BMJ Open* 2019;9(1):e024355. doi: 10.1136/bmjopen-2018-024355
9. Western B, Braga A, Hureau D, et al. Study retention as bias reduction in a hard-to-reach population. *Proceedings of the National Academy of Sciences* 2016;113(20):5477. doi: 10.1073/pnas.1604138113
10. Health Data Research UK. Health Data Research UK 2019 [Available from: www.hdruk.ac.uk.
11. CRIS. Clinical Records Interactive Search 2019 [Available from: https://www.maudsleybrc.nihr.ac.uk/facilities/clinical-record-interactive-search-cris/.
12. Farr Institute. The Farr Institute for Health Informatics Research 2019 [Available from: https://farrinstitute.org.
13. Gray L. The importance of post hoc approaches for overcoming non-response and attrition bias in population-sampled studies. *Social psychiatry and psychiatric epidemiology* 2016;51(1):155-57. doi: 10.1007/s00127-015-1153-8 [published Online First: 2015/11/28]
14. Norman JE, Morris C, Chalmers J. The effect of changing patterns of obstetric care in Scotland (1980-2004) on rates of preterm birth and its neonatal consequences: perinatal database study. *PLoS medicine* 2009;6(9):e1000153-e53. doi: 10.1371/journal.pmed.1000153 [published Online First: 2009/09/22]
15. Price A, Bryson H, Smith A, et al. Processes for engaging and retaining women who are experiencing adversity in longitudinal health services research. *BMC Health Serv Res* 2019;19(1):833-33. doi: 10.1186/s12913-019-4698-5

For peer review only - http://bmjopen.bmj.com/site/about/guidelines.xhtml

16. Aldhous MC, Bhatia R, Pollock R, et al. HPV infection and pre-term birth: a data-linkage study using Scottish Health Data. *Wellcome Open Res* 2019;4:48-48. doi: 10.12688/wellcomeopenres.15140.1
17. Hsieh DC, Smithers LG, Black M, et al. Implications of vaginal instrumental delivery for children's school achievement: A population-based linked administrative data study. *Australian and New Zealand Journal of Obstetrics and Gynaecology* 2019;59(5):677-83. doi: 10.1111/ajo.12952
18. Hann M, Roberts SA, D'Souza SW, et al. The growth of assisted reproductive treatment-conceived children from birth to 5 years: a national cohort study. *BMC Med* 2018;16(1):224-24. doi: 10.1186/s12916-018-1203-7
19. Martin P, Cortina-Borja M, Newburn M, et al. Timing of singleton births by onset of labour and mode of birth in NHS maternity units in England, 2005-2014: A study of linked birth registration, birth notification, and hospital episode data. *PloS one* 2018;13(6):e0198183-e83. doi: 10.1371/journal.pone.0198183
20. Thornton CE, Dahlen HG. Born before arrival in NSW, Australia (2000–2011): a linked population data study of incidence, location, associated factors and maternal and neonatal outcomes. *BMJ Open* 2018;8(3):e019328. doi: 10.1136/bmjopen-2017-019328
21. Harper G. Linkage of Maternity Hospital Episode Statistics data to birth registration and notification records for births in England 2005–2014: Quality assurance of linkage of routine data for singleton and multiple births. *BMJ Open* 2018;8(3):e017898. doi: 10.1136/bmjopen-2017-017898
22. Dattani N, Macfarlane A. Linkage of Maternity Hospital Episode Statistics data to birth registration and notification records for births in England 2005-2014: methods. A population-based birth cohort study. *BMJ open* 2018;8(2):e017897-e97. doi: 10.1136/bmjopen-2017-017897
23. Callander EJ, Fenwick J, Donnellan-Fernandez R, et al. Cost of maternity care to public hospitals: a first 1000-days perspective from Queensland. *Australian Health Review* 2019;43(5):556-64.
24. Minassian C, Williams R, Meeraus WH, et al. Methods to generate and validate a Pregnancy Register in the UK Clinical Practice Research Datalink primary care database. *Pharmacoepidemiology and Drug Safety* 2019;28(7):923-33. doi: 10.1002/pds.4811
25. ADR UK. Birth cohort data linkage study [Available from: https://www.adruk.org/our-mission/our-impact/article/birth-cohort-data-linkage-study-228/.
26. Howard LM, Piot P, Stein A. No health without perinatal mental health. *The Lancet* 2014;384(9956):1723-24. doi: 10.1016/S0140-6736(14)62040-7
27. Stein A, Pearson RM, Goodman SH, et al. Effects of perinatal mental disorders on the fetus and child. *The Lancet* 2014;384(9956):1800-19. doi: http://dx.doi.org/10.1016/S0140-6736(14)61277-0
28. Goldstein RF, Abell SK, Ranasinha S, et al. Association of Gestational Weight Gain With Maternal and Infant Outcomes: A Systematic Review and Meta-analysis. *JAMA* 2017;317(21):2207-25. doi: 10.1001/jama.2017.3635
29. Kahn RS, Zuckerman B, Bauchner H, et al. Women's Health After Pregnancy and Child Outcomes at Age 3 Years: A Prospective Cohort Study. *American Journal of Public Health* 2002;92(8):1312-18. doi: 10.2105/AJPH.92.8.1312

For peer review only

30. HRA. Health Research Authority. Section 251 and the Confidentiality Advisory Group (CAG) 2019 [Available from: http://www.hra.nhs.uk/about-the-hra/our-committees/section-251/ accessed 11 June 2019.
31. Stewart R, Soremekun M, Perera G, et al. The South London and Maudsley NHS Foundation Trust Biomedical Research Centre (SLAM BRC) case register: development and descriptive data. *BMC Psychiatry* 2009;9(1):51. doi: 10.1186/1471-244X-9-51
32. Fernandes AC, Cloete D, Broadbent MTM, et al. Development and evaluation of a de-identification procedure for a case register sourced from mental health electronic records. *BMC medical informatics and decision making* 2013;13:71-71. doi: 10.1186/1472-6947-13-71
33. Perera G, Broadbent M, Callard F, et al. Cohort profile of the South London and Maudsley NHS Foundation Trust Biomedical Research Centre (SLaM BRC) Case Register: current status and recent enhancement of an Electronic Mental Health Record-derived data resource. *BMJ Open* 2016;6(3):e008721. doi: 10.1136/bmjopen-2015-008721
34. Ristevski B, Chen M. Big Data Analytics in Medicine and Healthcare. *J Integr Bioinform* 2018;15(3):20170030. doi: 10.1515/jib-2017-0030
35. Zhang X, Pérez-Stable EJ, Bourne PE, et al. Big Data Science: Opportunities and Challenges to Address Minority Health and Health Disparities in the 21st Century. *Ethn Dis* 2017;27(2):95-106. doi: 10.18865/ed.27.2.95
36. CleverMed. BadgerNet Platform 2017 [Available from: https://www.clevermed.com/badgernet/.
37. Jewell A, Pritchard M, Barrett K, et al. The Maudsley Biomedical Research Centre (BRC) data linkage service user and carer advisory group: creating and sustaining a successful patient and public involvement group to guide research in a complex area. *Research Involvement and Engagement* 2019;5(1):20. doi: 10.1186/s40900-019-0152-4
38. NICE NIfH. Antenatal care for uncomplicated pregnancies 2008 [updated February 2019. Available from: https://www.nice.org.uk/guidance/cg62/chapter/1-Guidance2020.

Figure 1 - Data flow diagram for the eLIXIR Partnership linkage

For peer review only

For peer review only - http://bmjopen.bmj.com/site/about/guidelines.xhtml

The Data Flow Process

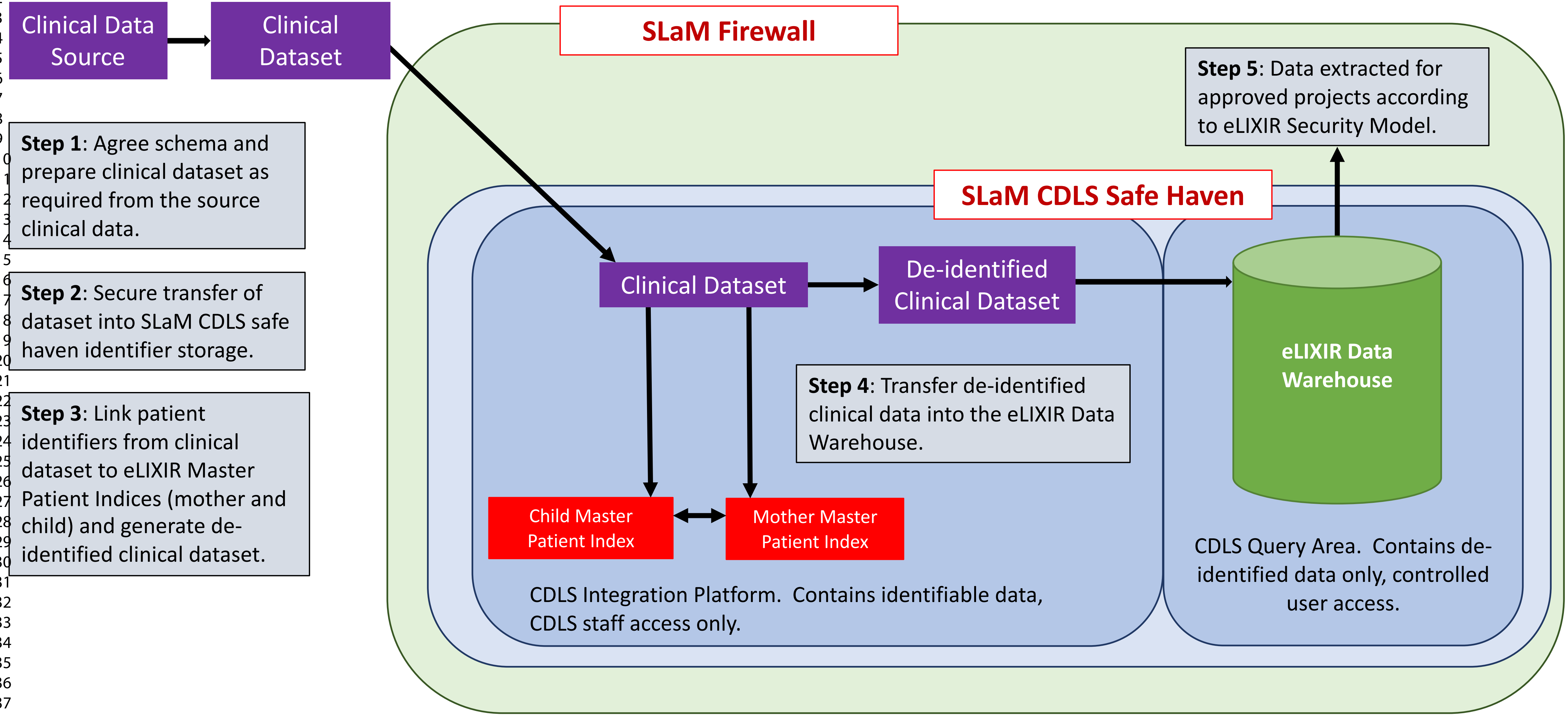

Supplement: Author's manuscript [file bmjopen-2020-039583.draft_revisions.pdf]
